# Systematic identification of p11 as a signaling modulator across the GPCRome

**DOI:** 10.1126/sciadv.aeg6567

**Published:** 2026-09-30

**Authors:** Marcus Saarinen, Ilana B. Kotliar, Inga Höffkes, Niclas Branzell, Carl-Fredrik Bowin, Leo Dahl, Elisa Da Silva, Vassilis Glaros, Alonso Abney, Annika Bendes, Taras Kreslavsky, Jochen M. Schwenk, Thomas P. Sakmar, Per Svenningsson

**Affiliations:** ^1^ Department of Clinical Neuroscience, Karolinska Institutet, Stockholm, Sweden.; ^2^ Science for Life Laboratory, School of Engineering Sciences in Chemistry, Biotechnology and Health, KTH Royal Institute of Technology, Solna, Sweden.; ^3^ Laboratory of Chemical Biology and Signal Transduction, The Rockefeller University, New York, NY, USA.; ^4^ Tri-Institutional PhD Program in Chemical Biology, New York, NY, USA.; ^5^ Department of Medicine Solna, Division of Immunology and Respiratory Medicine, Karolinska Institutet, Karolinska University Hospital, Stockholm, Sweden.; ^6^ Center for Molecular Medicine, Karolinska Institutet, Stockholm, Sweden.; ^7^ Division of Neurogeriatrics, Department of Neurobiology, Care Sciences and Society, Karolinska Institutet, Stockholm, Sweden.; ^8^ Basic and Clinical Neuroscience, King’s College London, London, UK.

## Abstract

The microprotein p11 (*S100A10*) is a ubiquitously expressed molecular scaffold that is known to be required for behavioral responses to certain antidepressants, presumably through its effect on G protein–coupled receptor (GPCR)–mediated signaling. Here, we demonstrate that p11 recruitment to GPCRs is promoted by an active receptor conformation. Furthermore, by mapping the GPCR-p11 interactome using a multiplexed suspension bead array (SBA) of 211 receptors, we identified more than two dozen high-confidence physical interactors across diverse GPCR signaling families. We define the functional significance of these interactions by focusing on a novel SBA screening hit, the protease-activated receptor 2 (PAR2). Transcriptomic fingerprinting and signaling assays in p11-knockout cells reveal that p11 acts as an amplifier of endogenous PAR2 signaling. PAR2 and p11 are specifically coexpressed in vivo in a subset of sensory neurons, and p11-deficient mice exhibit a blunted PAR2-mediated inflammatory response. Our results establish p11 as a widespread, activity-dependent modulator of GPCR-mediated signaling outcomes across diverse physiological processes.

## INTRODUCTION

G protein–coupled receptors (GPCRs) are the largest membrane receptor family in the human genome (*1*) and are targeted by approximately one-third of all Food and Drug Administration approved drugs (*2*). Primarily acting as signal transducers, GPCRs respond to a stimulus by modulating various intracellular signaling pathways, a process that is often specific to the receptor-ligand interaction its the cellular context (*3*). To orchestrate these responses, GPCRs interact with various cytoplasmic and membrane-associated proteins, such as heterotrimeric G proteins, GPCR kinases, β-arrestins, and other scaffold proteins including members of the 14-3-3 family (*4*, *5*). In addition, the receptor activity–modifying protein (RAMP) family regulates the function and pharmacology of a growing list of GPCRs (*6*). Accordingly, GPCR-RAMP assemblies have been exploited by a new class of migraine therapies targeting a complex between the GPCR calcitonin receptor–like receptor and RAMP1 (*7*), illustrating the importance of characterizing GPCR interactions to advance therapeutic interventions.

The S100 protein family comprises 24 members, among which S100A10, commonly known as p11 (*8*), has been reported to interact with a few GPCRs and ion channels using yeast-two hybrid and antibody (Ab) pull-down experiments (*9*–*13*). p11 is ubiquitously expressed in almost all cell types in the human body and can be induced or repressed by certain stimuli (*14*). For example, p11 brain mRNA levels are reduced in patients with major depressive disorder and can be reversed with electroconvulsive or antidepressant treatment (*9*). Furthermore, elevated p11 levels are correlated with multiple forms of cancer and are an unfavorable prognostic marker in breast, renal, lung, liver, head and neck, urothelial, and pancreatic cancer (*15*, *16*). The involvement of p11 in disease appears to be driven by its association with other proteins as a complex, as opposed to autonomous activity (*16*–*19*). For instance, p11 was found to interact with 5-hydroxytryptamine (5-HT) receptors 5-HT_1B/D_R and 5-HT_4_R (5-hydroxytryptamine receptor 4) and is required for the behavioral responses to antidepressant stimulation (*9*, *10*). Therefore, clarifying the context required for GPCR-p11 assembly and exploring interactions with other GPCRs would advance our comprehension of biological signaling networks regulating GPCR responses.

In this work, we show that p11 associates with 5-HT_4_R and that p11 is recruited to the surface plasma membrane (SPM) by receptor activation. To determine the interactome of p11 with a library of 211 GPCRs, we used a multiplexed suspension bead array (SBA) immuno-capture assay and found approximately two dozen GPCR-p11 interaction pairs that have not been previously reported. To investigate the impact and mechanism of GPCR-p11 coupling, we used both cellular and animal models to characterize the effect of the interaction of p11 with the protease-activated receptor 2 (PAR2). Overall, the results demonstrate the ability of p11 to form complexes with multiple GPCRs, which, in turn, can modulate GPCR signaling networks and cellular responses to ligand stimulation. This work highlights the importance of investigating GPCR interaction partners to further explore receptor biology and their associated signaling complexes.

## RESULTS

### GPCRs recruit p11 to the cell SPM

To investigate the mechanisms driving GPCR-p11 interactions, we first validated several control receptors using coimmunoprecipitation. On the basis of previous reports, p11 is known to associate with the 5-HT_4_R but not with the 5-HT_1A_R (*8*, *9*). We used dual epitope-tagged (DuET) (*20*) versions of these receptors, engineered to contain N-terminal FLAG and C-terminal 1D4 epitope tags, alongside a p11 construct containing tandem C-terminal OLLAS epitope tags. We then prepared solubilized membrane lysates from human embryonic kidney (HEK) 293T cells coexpressing DuET 5-HT_4_R or 5-HT_1A_R with p11-OLLAS and immunoprecipitated the lysates with a 1D4 monoclonal antibody (mAb). We immunoblotted against p11-OLLAS to reveal a lysate dose-dependent and 1D4-specific co-pulldown of p11 with 5-HT_4_R, confirming the expected 5-HT_4_R-p11 interaction (Fig. 1A and fig. S1). In contrast, no signal was detected in samples expressing DuET 5-HT_1A_R or using unspecific immunoglobulin G (IgG) for the pull-down, establishing these receptors as appropriate positive and negative controls for further investigation of GPCR-p11 interactions.

**Fig. 1.**
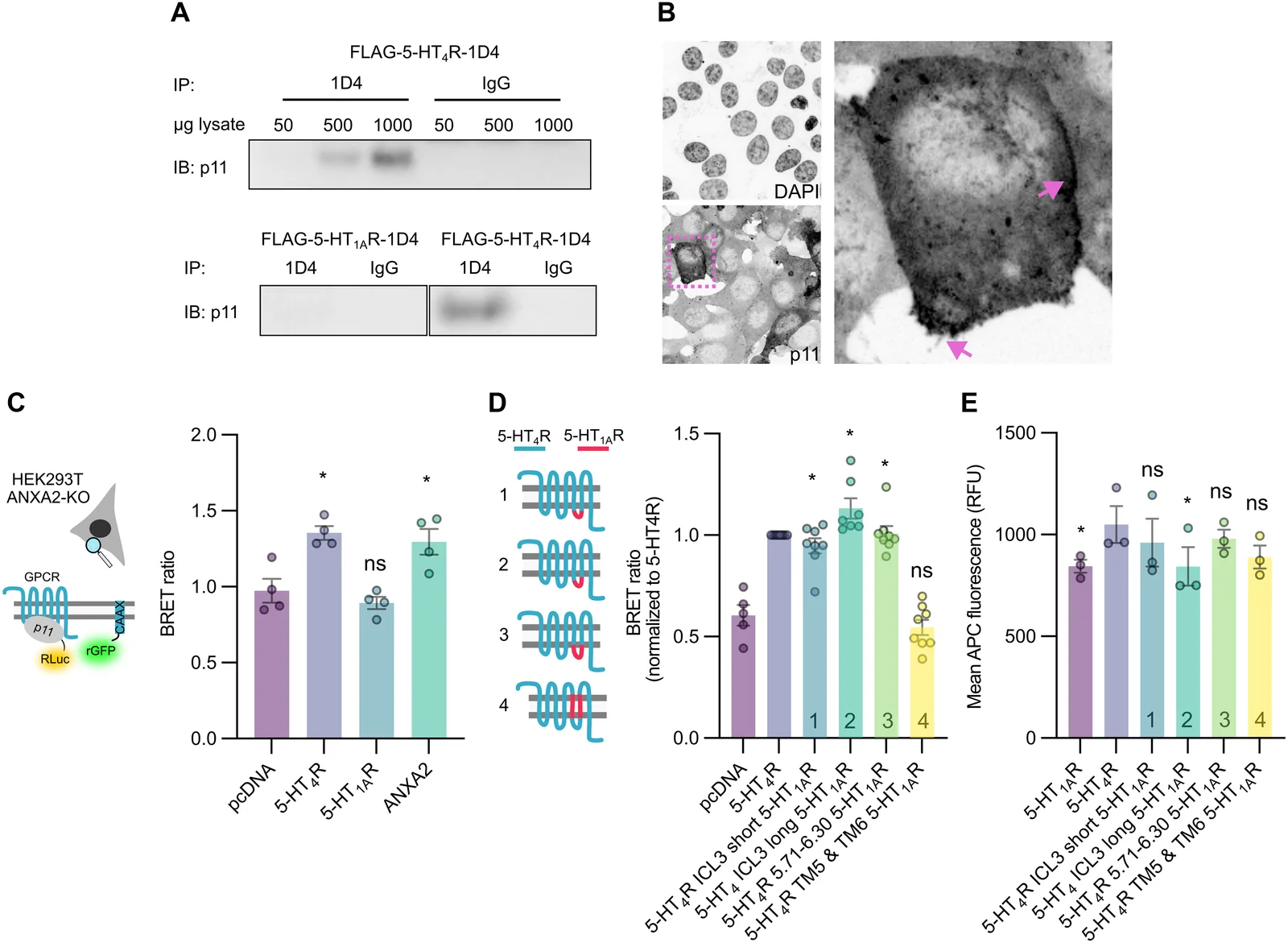
GPCRs recruit p11 to the SPM. (**A**) Top: HEK293T cells were cotransfected with FLAG-5HT_4_R-1D4 and p11-OLLAS, followed by immunoprecipitation using increasing amounts of purified membrane lysate with either 1D4 or IgG antibodies. Immunoprecipitated proteins were subsequently analyzed via Western blotting using antibodies against p11. Bottom: Western blot targeting p11 on 1D4-immunoprecipitated, membrane-solubilized samples coexpressing p11-OLLAS and DuET 5-HT_1A_R or 5-HT_4_R. Uncropped images are available in the Supplementary Materials. (**B**) Endogenous p11 membrane localization in HEK293T cells. Two-dimensional view of DAPI (upper-left) and p11 (bottom-left) in S100A10-OpenCell HEK293T cells. Image is adapted from the online OpenCell database. Larger panel on the right is an expansion of the p11-channel, highlighted with a magenta square. Membranous p11 is highlighted with arrows. (**C**) Left: overview of p11 membrane recruitment assay via ebBRET. RLuc (bright orange) tagged p11 is coexpressed together with a DuET GPCR and CAAX-rGFP (green) to monitor p11 membrane recruitment. Right: ebBRET results from coexpressing p11-RLuc with CAAX-rGFP and DuET 5-HT_4_R, 5-HT_1A_R, FLAG-ANXA2, or empty pcDNA [*P < 0.05, comparison to pcDNA, one-way analysis of variance (ANOVA), Dunnet’s multiple comparisons test]. (**D**) Overview of chimeric receptors for measuring p11 membrane recruitment of various 5-HTR constructs. ebBRET ratios normalized to WT 5-HT_4_R (*P < 0.05, comparison to pcDNA, one-way ANOVA, Dunnet’s multiple comparisons test). (**E**) Bar plots depicting surface expression of various 5-HTR constructs related to Fig. 1D. *Y* axis represents measured APC mean fluorescence intensity (MFI) for each sample with each dot representing an independent sample (*P < 0.05, comparison to 5-HT_4_R, one-way ANOVA, Dunnet’s multiple comparisons test). All points represent individual experiments with error bars indicating mean ± SEM. ns, not significant.

Next, we used CRISPR technology to generate monoclonal p11 knockout (KO) and annexin-A2 (ANXA2) KO HEK293T cell lines (fig. S2) to establish an appropriate cellular context for evaluating these interactions. The ANXA2-KO line was specifically designed to resolve whether GPCR-p11 interactions are dependent on the heterotetrameric ANXA2-p11 scaffold, which is traditionally required for the stability and cell surface localization of p11 (*21*, *22*). Likewise, we sought to clarify the spatial context of these interactions by characterizing the localization of endogenous p11. Using the OpenCell database (*23*) and the split-mNeonGreen2 endogenous tagging system, we confirmed that endogenous p11 is localized to the cell SPM (Fig. 1B). To determine whether specific GPCRs can actively recruit p11 to this compartment, we established an enhanced bystander BRET (ebBRET) assay system in ANXA2-KO HEK293T cells by coexpression of RLucII-tagged p11 (p11-RLuc) with membrane-tethered rGFP (rGFP-CAAX), to monitor p11 recruitment in real time. We observed significant SPM recruitment of p11 in the presence of 5-HT_4_R or the positive control ANXA2, while recruitment levels in 5-HT_1A_R–expressing cells were indistinguishable from those of empty pcDNA controls (Fig. 1C). These results indicate that the 5-HT_4_R can drive p11 SPM recruitment, even in the absence of ANXA2.

Last, to identify the minimal structural motif sufficient for this recruitment, we generated a series of 5-HT_4_R/5-HT_1A_R chimeras, targeting segments previously implicated in p11 binding, such as transmembrane helix 5 (TM5), TM6, and intracellular loop 3 (ICL3). While swapping the ICL3 of 5-HT_4_R for that of 5-HT_1A_R did not impair p11 recruitment, chimeric constructs replacing the TM5/6 helical bundle resulted in a significant loss of recruitment (Fig. 1D). To ensure that these differences were not due to impaired receptor trafficking, cell surface expression for all chimeras was independently verified via flow cytometry (Fig. 1E and fig. S3).

### Constitutive activity drives p11 recruitment

To clarify the context driving the SPM recruitment of p11, it was necessary to quantitate direct GPCR-p11 interactions. To do so, we developed a BRET assay using C-terminally tagged GPCR-NLuc as the donor and p11-mVenus as the acceptor. We first validated this platform in p11-KO cells, observing a fourfold higher netBRET signal upon coexpression of p11-mVenus with 5-HT_4_R-NLuc compared to 5-HT_1A_R–NLuc (fig. S4). Replicating these experiments in ANXA2-KO cells yielded nearly identical results, confirming that the 5-HT_4_R-p11 interaction is detectable in our system and can occur independently of the ANXA2 scaffold. Next, we constructed chimeric receptors where the TM5 and TM6 segments of the p11 interacting 5-HT_4_R were individually swapped into the noninteracting 5-HT_1A_R backbone (Fig. 2A). Replacing the TM5 of 5-HT_1A_R with that of 5-HT_4_R resulted in a significant increase in netBRET compared with wild-type (WT) 5-HT_1A_R. Conversely, no such enhancement was observed with the TM6 chimeric construct, indicating that the residues driving physical recruitment reside primarily within the TM5 domain. Beyond recruitment, we observed that interactions driven by specific receptor domains alter p11-mVenus protein abundance in our cotransfection system; p11-mVenus fluorescence was higher when coexpressed with 5-HT_4_R-NLuc (15-fold) or the TM5 chimera (5-fold), but not with 5-HT_1A_R–NLuc or the TM6 chimera compared to no receptor (fig. S5, A and B). To determine whether this increase was specific, we titrated p11-mVenus against a stable gene dose of the coexpressed receptor. In the presence of 5-HT_4_R or the TM5 chimera, both the rate (slope) and maximal p11-mVenus abundance were significantly higher than in the presence of 5-HT_1A_R or the TM6 chimera (Fig. 2B).

**Fig. 2.**
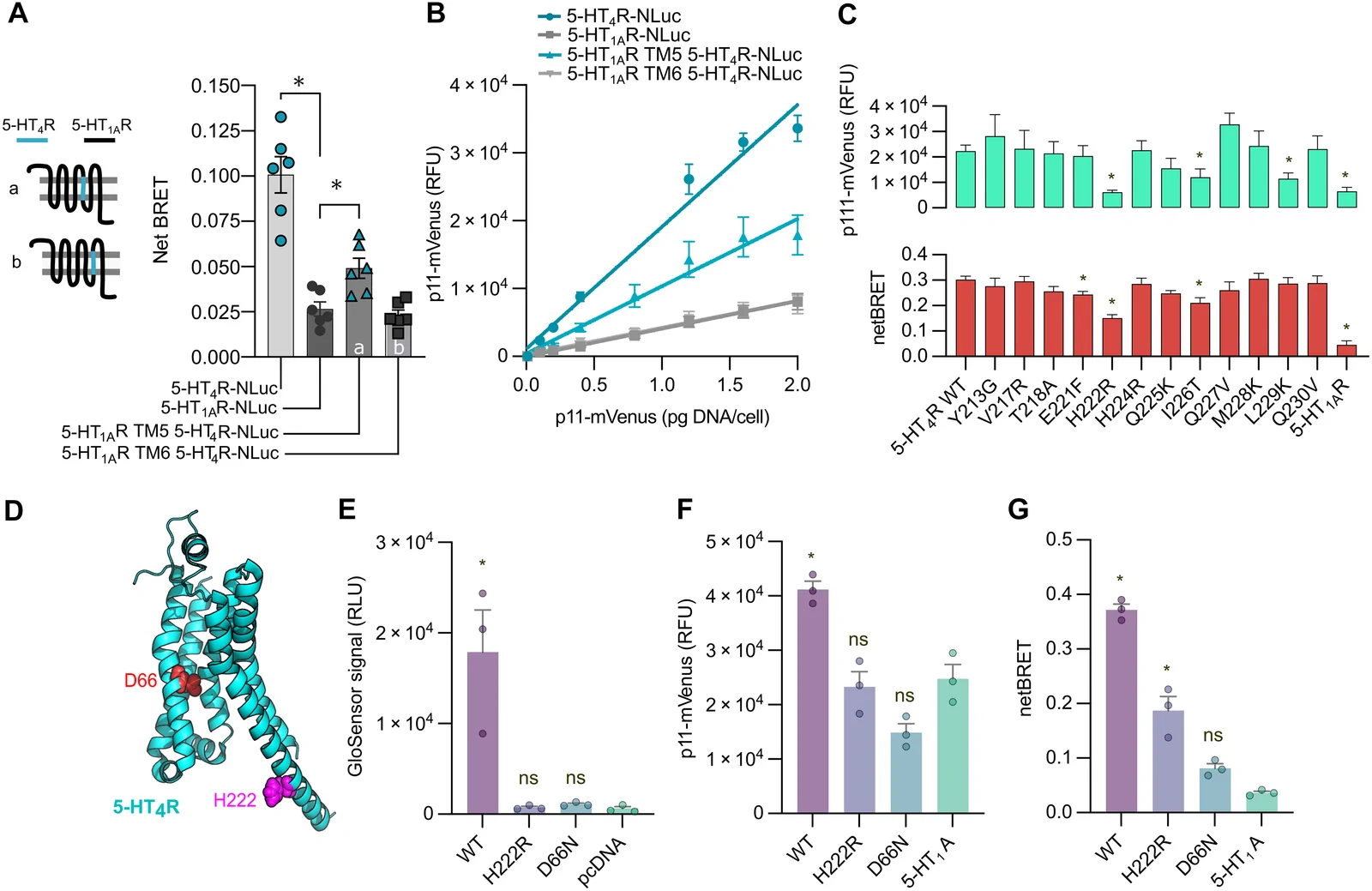
p11 is recruited by constitutively active 5-HT_4_R. (**A**) Left: overview of chimeric constructs replacing either TM5 or TM6 of 5-HT_1A_R with that of 5-HT_4_R. Right: netBRET of constructs when coexpressed with p11-mVenus (0.1 pg receptor DNA per cell, 1 pg p11-mVenus DNA per cell) (*P < 0.05 construct versus 5-HT_1A_R–NLuc, one-way ANOVA with Dunnet’s multiple comparisons test). (**B**) p11-mVenus fluorescence counts when increasing p11-mVenus gene dose and cotransfection with a stable gene dose of receptor (0.02 pg DNA per cell, *n* = 4) [slopes of the plotted lines: 5-HT_4_R (17995), 5-HT_1A_R (4081), TM5 chimera (9930), and TM6 chimera (3972)]. (**C**) Top: p11-mVenus levels (measured before BRET readings) upon coexpression with 5-HT_4_R-NLuc receptor mutants. Bottom: netBRET between p11-mVenus and coexpressed 5-HT_4_R-NLuc receptor mutants (*P < 0.05 construct versus 5-HT_4_R WT, one-way ANOVA with Dunnet’s multiple comparisons test) (*n* = 3 to 6). (**D**) Position of H222 (magenta) and D66 (red) in a cryo–electron microscopy structure of 5-HT_4_R (PDB: 7XT9, cyan). Some transmembrane components have been removed for clarity. The intracellular surface is toward the bottom of the figure. (**E**) cAMP sensor (0.2 pg DNA per cell) signal when coexpressed with either 5-HT_4_R WT-NLuc, 5-HT_4_R H222R-NLuc, or 5-HT_4_R D66N-NLuc (0.08 pg DNA per cell) without ligand stimulation. (*P < 0.05, comparison to pcDNA, one-way ANOVA, Dunnet’s multiple comparisons test). (**F**) p11-mVenus fluorescence levels in p11-KO cells coexpressing p11-mVenus (1 pg DNA per cell) and 5-HT_4_R WT-NLuc, 5-HT_4_R H222R-NLuc, or 5-HT_4_R D66N-NLuc (0.002 pg DNA per cell). (**G**) netBRET of p11-mVenus (1 pg DNA per cell) coexpressed with 5-HT_4_R WT-NLuc, 5-HT_4_R H222R-NLuc, or 5-HT_4_R D66N-NLuc (0.002 pg DNA per cell). All points represent individual experiments with error bars indicating mean ± SEM.

Next, we measured cyclic adenosine 3′,5′-monophosphate (cAMP) signaling in cells transfected with the chimeric constructs to determine whether a gain of function in the TM5 chimera contributed to its enhanced association and impact on p11 (*24*). We observed both elevated basal and ligand-stimulated cAMP with the TM5 chimera, but not with the TM6 chimera (fig. S5C). This functional shift suggests that the observed accumulation of p11-mVenus in our cotransfection system may be linked to increased basal activity or receptor-mediated Gα_s_ signaling. Furthermore, a 4-hour treatment with either the adenylyl cyclase (AC) stimulator forskolin or the AC inhibitors 2′,5′-dideoxyadenosine and SQ22536 had no effect on the netBRET signal for any construct, despite a surge in p11-mVenus levels with forskolin treatment (fig. S6). To identify specific residues within TM5 driving the p11 interaction, we performed a scanning mutagenesis screen, replacing 12 residues in the 5-HT_4_R TM5 domain with their counterparts from the noninteracting 5-HT_1A_R. We identified three mutations—E221F, H222R, and I226T—that significantly decreased netBRET compared to the WT receptor (Fig. 2C). Three mutations (H222R, I226T, and L229K) also reduced p11-mVenus protein levels. To provide structural insight complementing our mutagenesis findings, we modeled the p11/5-HT4R complex using AlphaFold 3 (AF3). Although interface prediction confidence was low [interface predicted template modeling (iPTM) scores, ∼0.25 to 0.30], the resulting model is consistent with our biochemical data, localizing a putative p11 binding interface to the intracellular boundary of TM5 (fig. S7). This predicted surface encompasses the specific intracellular residues identified in our functional assays. We prioritized H222R for further study because H222 is located on the intracellular face of TM5 in a position of direct contact with the Gα_s_ subunit (*24*). Therefore, we hypothesized that it would impair Gα_s_-mediated signaling by preventing Gα_s_ docking while still allowing the receptor to sample an active conformation.

To test whether p11 recruitment depends on downstream signaling or receptor conformation, we compared H222R with the D66N (D^2.50^N) mutant, which we predicted would discourage constitutive G protein interactions through a distortion of the NPxxY microswitch (*25*). While both H222R and D66N failed to generate basal cAMP or increase p11-mVenus abundance (Fig. 2, E and F), they exhibited distinct BRET profiles. The H222R mutant maintained a significantly higher basal BRET (∼0.19) than the 5-HT_1A_R control (∼0.05), whereas the inactive D66N mutant showed only a slightly elevated signal (∼0.08) (Fig. 2G). Both mutants could be activated by 5-HT, which also resulted in increased p11-mVenus protein abundance and complex formation, although with markedly reduced potencies (fig. S8, A to C). Last, we confirmed that 5-HT_4_R constitutive signaling could be inhibited by the inverse agonist GR 125487 (fig. S8D) (*26*). Correspondingly, overnight treatment with GR 125487 induced a concentration-dependent decrease in both p11-mVenus protein levels and its recruitment to 5-HT_4_R (fig. S8, E and F).

Pharmacological assays confirmed that p11-mVenus undergoes constitutive proteasomal degradation, as evidenced by increased protein levels following overnight treatment with the proteasomal inhibitor MG132 (fig. S9A). However, direct activation of AC with overnight Forskolin treatment induced a significantly greater accumulation of p11-mVenus, suggesting that in our system, the cellular p11-mVenus pool is highly sensitive to cAMP-mediated signaling. This relationship aligns with the kinetics of p11-mVenus recruitment in our assay; in overnight recordings, the BRET signal for agonist-stimulated 5-HT_4_R-D66N and p11-mVenus only diverged from vehicle controls ∼200 min poststimulation (fig. S9B). This delay coincides with the window required for signaling-driven protein accumulation, suggesting that the assay captures the progressive accumulation of receptor-p11 complexes rather than immediate physical recruitment, which may be below the detection limit or subject to rapid initial turnover. Last, we confirmed that this accumulation does not inherently lead to false-positive signals. Even when we artificially drove p11-mVenus to high levels using a trans-activation approach (cotransfecting 5-HT_1A_R–NLuc with DuET 5-HT_4_R), the direct 5-HT_4_R-NLuc-p11-mVenus interaction generated a netBRET signal significantly higher than this signaling-induced bystander noise (fig. S9C). Furthermore, we repeated netBRET measurements by cotransfecting p11-KO or ANXA2-KO cells with either 5-HT_4_R-NLuc/p11-mVenus or 5-HT_1A_R–NLuc/p11–mVenus, and both conditions showed a similar netBRET, despite a larger accumulation of p11-mVenus in 5-HT_4_R-NLuc-p11-mVenus–transfected p11-KO cells than in ANXA2-KO cells (fig. S10, A to D). Collectively, these data indicate that while receptor-mediated signaling can expand the cellular p11-mVenus pool in our cotransfection system, the specificity of the physical recruitment, at least in the case of the 5-HT_4_R, appears to be governed by the receptor’s conformational state.

### A multiplexed screen identifies numerous new GPCR-p11 interactions

To determine whether p11 can interact with more GPCRs than previously described, we designed a multiplexed SBA to screen a library of 211 DuET human GPCRs (Fig. 3A). This platform was chosen because it uses solubilized membrane lysates, enabling the detection of direct protein-protein complexes. This approach was favorable, since it would allow for us to distinguish receptors that physically engage p11 from those that just increase its cellular abundance through downstream activity. To do so, we used the p11-OLLAS bait construct and coexpressed it individually with our library of DuET GPCRs in Expi293F cells. We incorporated multiple controls, including GPCR samples without p11-OLLAS, mock-transfected cells, and p11-OLLAS only samples, to perform quality control and establish statistically robust *z*-scores based on mean fluorescence intensity (MFI) (fig. S11, A and B). While multiple capture-detection schemes were initially tested, we focused our analysis on FLAG capture-OLLAS detection and 1D4 capture-OLLAS detection due to their effective discrimination between controls. This approach yielded 44 and 42 positive hits, respectively, with 25 unique double hits appearing in both independent capture datasets (Fig. 3B and fig. S11C).

**Fig. 3.**
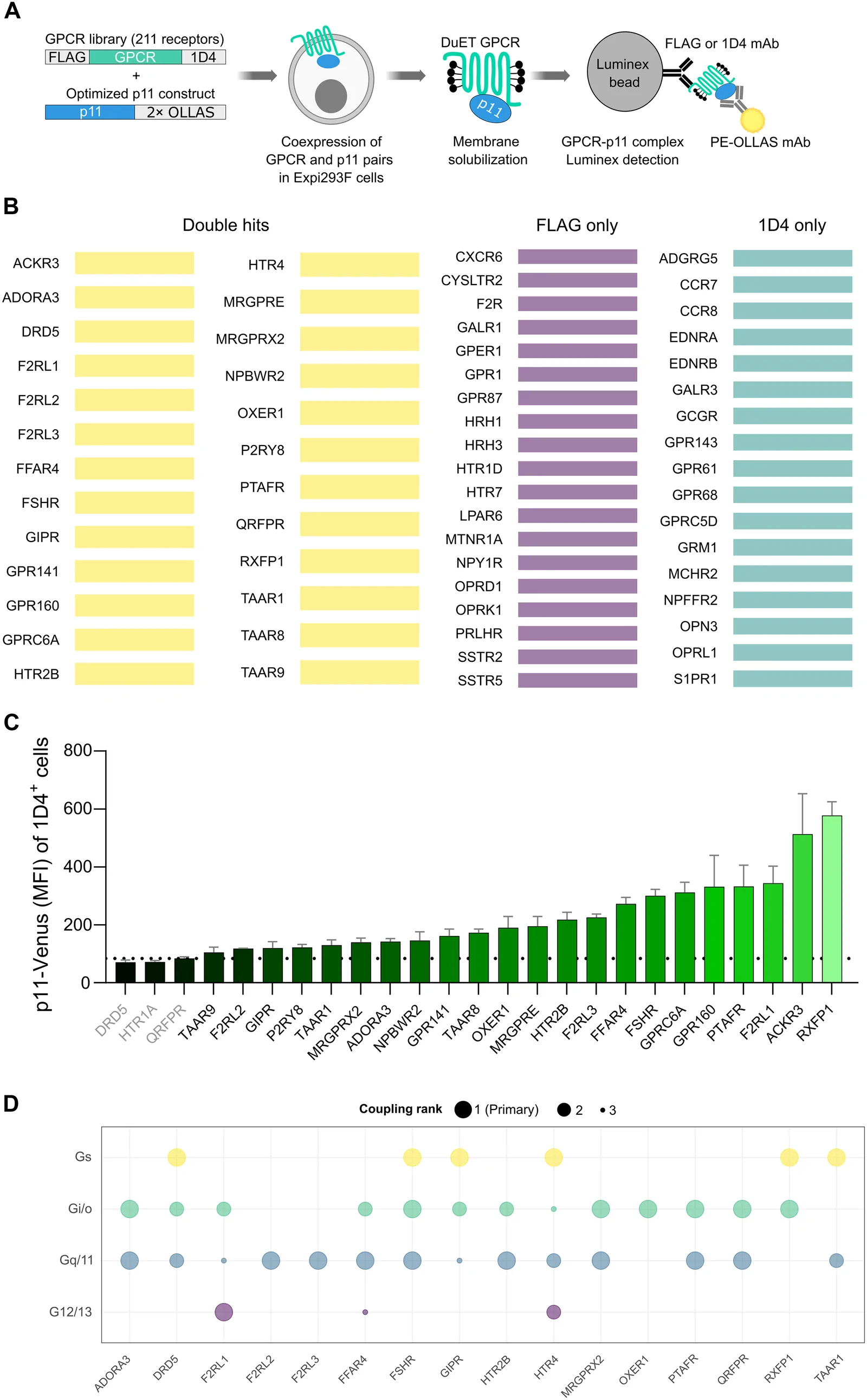
A map of GPCR-p11 interactions. (**A**) Visualization of the SBA experimental setup where membrane proteins from cells coexpressing DuET GPCRs and p11-OLLAS are solubilized, isolated, and incubated with Ab-linked Luminex beads. Subsequently, beads are washed to remove unbound lysate, and the protein complexes are detected using phycoerythrin (PE)–conjugated Abs. Two capture-detection schemes were used: FLAG capture-OLLAS detection or ID4 capture-OLLS detection. (**B**) Bar code map of all DuET GPCRs found to interact with p11-OLLAS, colored by the number of capture-detection schemes they passed. (**C**) Bar graph depicting p11-mVenus MFI across various receptor (1 pg DNA per cell) and p11-mVenus (1 pg DNA per cell) pairs. Each bar signifies a distinct receptor-p11 pair and represents MFI of the receptor and p11 positive cell population measured from three independent samples. Grayed-out receptor gene names indicate those that failed to pass the statistical cutoff, which is depicted as a horizontal dotted line. Error bars indicate mean ± SEM. (**D**) G protein coupling profiles available for selected double hits from the primary screen, with dot size increasing with coupling priority. Sourced from gpcrdb.org.

Next, to ensure that hits were not biased by variable GPCR expression levels, the MFI values of FLAG and 1D4 capture-detection schemes across hits and nonhits were compared. No significant differences were found between the groups, indicating that the hits were not likely related to receptor expression levels (fig. S11D). Conversely, across both single and double hits, we observed a significant increase in mean p11-OLLAS levels in the hit samples compared with nonhit samples. High p11 expression alone was not enough for hit classification, since dozens of nonhits were also associated with similar p11-OLLAS protein levels as hits. The full screening results are provided in fig. S12. To validate whether these SBA hits truly elevate p11 protein levels upon coexpression, we used two orthogonal assays in p11-KO HEK293T cells. We first coexpressed p11-mVenus with all 24 double-positive hits and the 5-HT_4_R control, measuring total mVenus fluorescence corrected for cell density in microwell plates. At a p11:GPCR gene dosage ratio of 1:5, 9 of the 24 hits exhibited higher p11-mVenus signals than the 5-HT_1A_R negative control (mean + 3*SD of 5-HT_1A_R) (fig. S13). Increasing this ratio to 1:1 resulted in 16 of the 24 complexes surpassing the statistical hit threshold, indicating that p11-mVenus levels vary with the abundance of the cotransfected GPCR.

However, recognizing that total fluorescence measurements from a single well do not capture the heterogeneity of receptor expression inherent in transient transfections, we used flow cytometry to quantitate both DuET GPCR and p11-mVenus abundance. To validate the feasibility of this approach, we initially cotransfected p11-mVenus with FLAG-tagged ANXA2, observing a clear linear relationship between ANXA2 abundance and p11 levels (fig. S14A). Subsequent flow cytometry of our DuET GPCR hits and p11-mVenus at a 1:1 gene ratio revealed that p11-mVenus expression was strictly dependent on the abundance of the interacting receptor. For example, the expression of the novel hit RXFP1 strongly correlated with p11 coexpression, whereas the noninteracting 5-HT_1A_R did not (fig. S14B). Collectively, these single-cell readouts confirmed that 22 of the 25 GPCRs identified in the SBA screen induce significant increases in cellular levels of coexpressed p11-mVenus (Fig. 3C).

To verify that the increase in p11-mVenus levels observed in our hits was not only a nonspecific artifact of cAMP-mediated promoter stimulation, we first tested whether Gα_s_ activation could drive the accumulation of other mVenus-tagged control constructs. We stimulated the Gα_s_-coupled double-hit TAAR1 with its agonist D-amphetamine and compared the response of p11-mVenus to that of control RAMP1/2/3-mVenus constructs. While TAAR1 stimulation produced a modest increase in RAMP-mVenus levels, this effect was much lower in magnitude than the accumulation observed for p11-mVenus (fig. S15, A and B). Likewise, an analysis of the G protein–coupling profiles for the 25 SBA double hits revealed a diverse signaling landscape spanning primary coupling to Gα_i/o_, Gα_q/11_, and Gα_12/13_ as well as Gα_s_ (Fig. 3D). The diversity of the hits observed here suggests that p11 engagement is a widespread feature across the GPCR superfamily and led us to prioritize the protease-activated receptor 2 (PAR2/F2RL1), a receptor with broad, non-Gα_s_ coupling, for further functional characterization.

### Modulation of endogenous PAR2 signaling by p11

We chose to characterize the effects of p11 presence on PAR2 function for three primary reasons. First, it was identified as a robust double hit in our multiplexed SBA screen. Second, we validated that the receptor is endogenously expressed in HEK293T cells, providing a native context for study (fig. S16). Third, it is a valid therapeutic target for treating pain and inflammatory conditions (*27*, *28*). To further evaluate the association between PAR2 and p11 complexes, we investigated whether PAR2 activation might influence the spatial distribution of p11. Using the S100A10-OpenCell HEK293T cell line to track endogenous p11, we noted colocalization of PAR2-mScarlet3 and p11 at the SPM (Fig. 4A). Quantitating the SPM-localized signal indicated an agonist-driven increase of endogenous p11 at the membrane following stimulation with the PAR2 agonist 2-Furoyl-LIGRLO-amide in PAR2-mScarlet3–transfected cells (Fig. 4B). This effect appeared specific to PAR2, as agonist stimulation of the noninteracting 5-HT_1A_R–mScarlet control did not significantly alter endogenous p11 membrane accumulation.

**Fig. 4.**
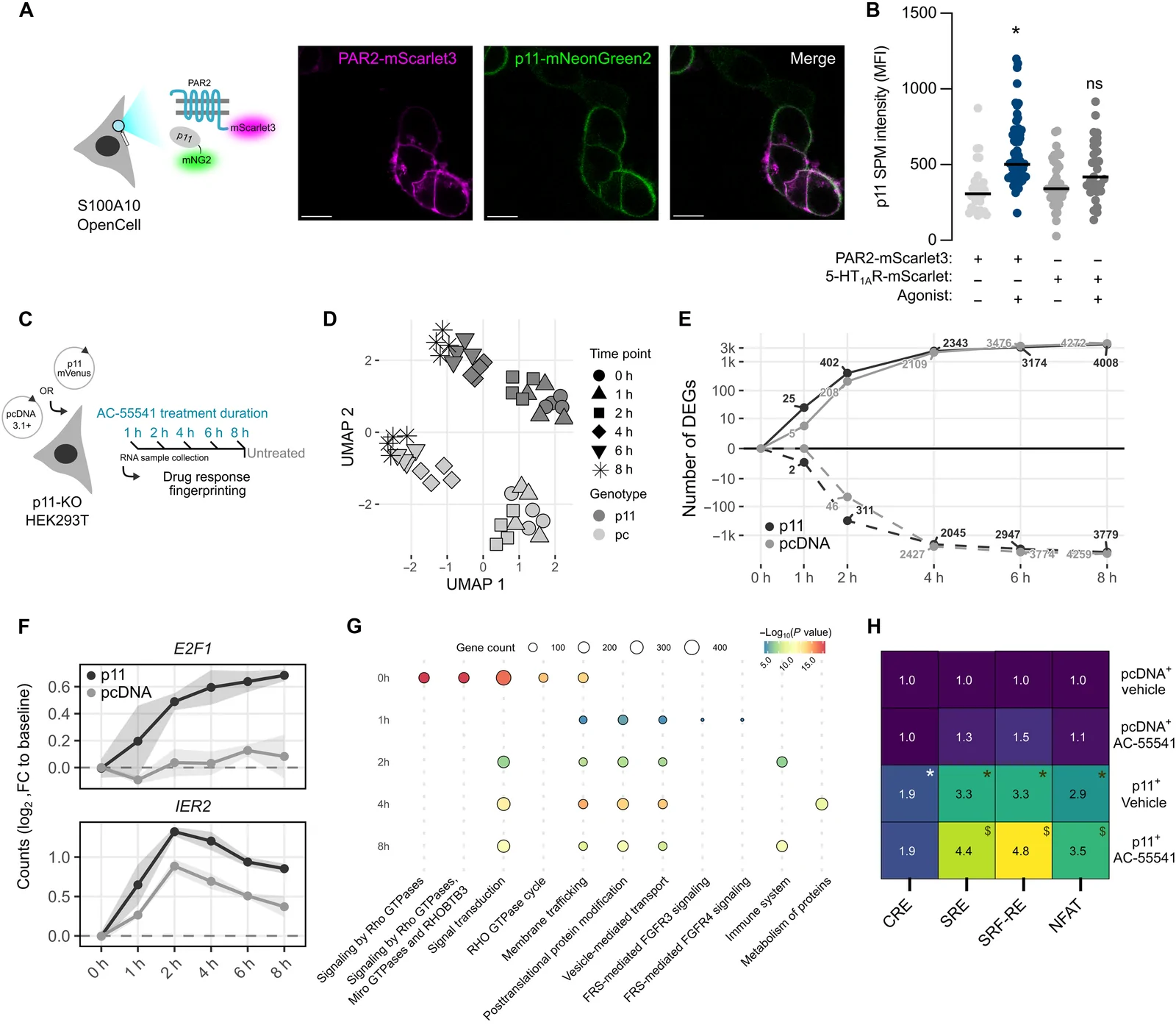
p11 alters the sensitivity of PAR2 signaling. (**A**) Active PAR2 colocalizes with endogenous p11 on the SPM in S100A10-OpenCell cells transfected with PAR2-mScarlet3 [representative images from (B); scale bar: 10 μm, *n* = 3]. (**B**) Quantitation of endogenous p11 recruitment to the SPM in cells cotransfected with PAR2-mScarlet3 (±10 μM 2-Furoyl-LIGRLO-amide, 1 hour) or 5-HT1AR–mScarlet (±5-HT, 1 hour) (*P < 0.05 agonist versus vehicle, ns *P* > 0.05, two-way ANOVA with Tukey’s test). (**C**) Overview of the experimental approach to determining transcriptome-wide changes of endogenous PAR2 signaling in HEK293T cells, in the presence or absence of p11. Eight-hour samples were treated first, and 1-hour samples were treated last. All samples were collected at the same time point (*n* = 4). (**D**) UMAP of each pcDNA or p11-mVenus–transfected sample for each AC-55541 treatment time point. (**E**) Line plot of the number of DEGs in response to AC-55541 for pcDNA and p11-mVenus groups. Significant DEGs were calculated on the basis of each groups own baseline (no treatment). (**F**) Raw counts (shown as a log_2_ fold change to the groups own baseline) of two representative IEGs found to be more responsive to PAR2 activation in p11-mVenus–transfected samples. (**G**) Top five significant Reactome pathways up-regulated in p11-mVenus versus pcDNA cells across treatment time points. GTPases, guanosine triphosphatases. (**H**) Validation of RNA-seq results using response element–driven luciferase constructs (1 pg DNA per cell) cotransfected with either p11-mVenus or pcDNA controls (1 pg DNA per cell). Heat plot shows the fold change of the measured luminescence from AC-55541– or dimethyl sulfoxide (DMSO)–treated samples relative to pcDNA DMSO–treated controls (*n* = 6 to 8 biological replicates, *P < 0.05 p11 vehicle versus pcDNA AC-55541, $*P* < 0.05 p11 AC-55541 versus p11 vehicle, two-way ANOVA with uncorrected Fishers LSD). h, hours.

Building on these observations suggesting that PAR2 activation promotes p11 membrane localization, we next sought to assess the broader influence of p11 on native PAR2 signaling. We performed RNA sequencing (RNA-seq) on p11-KO HEK293T cells rescued with either p11-mVenus or an empty vector (Fig. 4C). This strategy enabled us to characterize the response of endogenous PAR2 to a saturating dose of the specific PAR2 agonist AC-55541 (fig. S17) (*29*) at various time points. Comparison of drug naive transcriptomes revealed that p11-mVenus expression alone exerts a profound effect on the cellular landscape, with more than 4000 differentially expressed genes (DEGs) identified between the genotypes (fig. S18 and table S1). UMAP clustering of the untreated and AC-55541–stimulated samples confirmed a transcriptomic response to the drug, with both genotypes following temporally unique trajectories (Fig. 4D). Specifically, p11-mVenus–expressing cells showed a more distinct separation of the early 1- to 2-hour clusters on the UMAP, whereas both genotypes clearly diverged from the 4-hour time point onward. This accelerated transcriptomic shift was confirmed by DEG analysis; at 1 and 2 hours posttreatment, p11-mVenus samples exhibited a stronger response than controls (25 versus 5 and 402 versus 208 DEGs, respectively) (Fig. 4E and table S1). Many genes driving this early divergence are known immediate early genes and representative genes, such as *E2F1* and *IER2*, exhibited accelerated and more pronounced induction in the presence of p11 than in its absence (Fig. 4F). Since our p11-mVenus plasmid encodes the human p11 RNA sequence, it was detected and quantitated in the RNA-seq data (fig. S18), confirming a strong overexpression of *S100A10* in each p11-mVenus–transfected sample. *S100A10* was significantly up-regulated in response to AC-55541 treatment only in p11-mVenus–transfected samples (fig. S19).

To explore the functional networks affected by p11, we analyzed the top five most significant Reactome pathways up-regulated in p11-mVenus–transfected cells relative to pcDNA controls across the time course. This analysis allowed us to distinguish between biological processes enriched at baseline and those specifically induced by AC-55541 (Fig. 4G). We observed that genes involved in certain pathways, such as vesicle-mediated transport, became significantly up-regulated only starting after AC-55541 treatment. In contrast, genes involved in membrane trafficking and signal transduction pathways were enriched at baseline and remained elevated throughout the time course. Genes involved in Rho guanosine triphosphatase signaling exhibited the strongest enrichment at baseline, with a comparatively lower magnitude of enrichment following ligand stimulation.

To validate these transcriptomic findings and map the specific G protein outputs of endogenous PAR2 in our system, we used response element–driven luciferase assays in p11-KO HEK293T cells (Fig. 4H). We cotransfected luciferase reporters for NFAT (Gα_q_), SRE (Gα_i_), SRF-RE (Gα_12/13_), or CRE (Gα_s_) alongside either p11-mVenus or pcDNA. Consistent with the known signaling profile of PAR2, stimulation with AC-55541 induced responses in the NFAT, SRE, and SRF-RE reporters, whereas the CRE reporter remained nonresponsive to the agonist. Notably, cells expressing p11-mVenus exhibited significantly higher baseline luciferase activity across all reporters compared to pcDNA controls, corroborating the elevated signal transduction signatures identified in the RNA-seq dataset. Furthermore, the presence of p11-mVenus significantly increased the magnitude of the response to AC-55541 across the Gα_q_, Gα_i_, and Gα_12/13_ pathways. This lack of response in the CRE reporter further confirms that the agonist-induced effects were specific to the known G protein coupling of PAR2 and were not a result of nonspecific signaling cross-talk (*30*). Further analysis indicated that this amplification likely results from greater PAR2 surface levels following agonist treatment with p11 than in the absence of p11 (fig. S20A). p11 appears to maintain the active receptor on the SPM, rather than regulating the constitutive forward trafficking of ligand naive PAR2, which would result in globally shifted or pronounced baseline responses, a phenomenon which we did not observe (fig. S20, B and C).

To determine whether p11 modulates PAR2-mediated physiological responses in vivo, we sought to identify a quantitative assay of PAR2 activation. Previous studies have demonstrated that subcutaneous injection of PAR2 agonists induces significant hind paw swelling, a response attributed to a specific subset of dorsal root ganglion (DRG) neurons (Fig. 5A) (*31*, *32*). To evaluate p11 involvement in this circuit, we analyzed a deeply sequenced mouse single-cell RNA sequencing (scRNA-seq) dataset to map the expression of *S100a10* (p11) and *F2rl1* (PAR2) (*33*). Our analysis of the scRNA-seq data confirmed that p11 is abundantly expressed throughout the DRG, including in the specific cluster of PAR2-expressing neurons characterized by the markers such as *Nppb* (Fig. 5B). To evaluate the physiological impact of this coexpression, we used a global p11-KO mouse model to quantify the inflammatory response to PAR2 activation. Specifically, p11-WT and p11-KO mice received subcutaneous injections of either saline or AC-55541. Paw thickness was measured 1 hour postinjection followed by 30-min intervals to monitor the progression of edema. Both treatments caused an inflammatory response (Fig. 5C), peaking at 1 hour postinjection. We observed significant inflammation caused by AC-55541 compared to the vehicle treatment in both p11-WT and in p11-KO mice (Fig. 5D, top). However, further analysis of the inflammation specifically evoked by AC-55541 revealed a significantly dampened response in p11-KO mice compared to the p11-WT mice (Fig. 5D, bottom). To ensure that the observed reduction in inflammatory response was specifically linked to PAR2 signaling rather than a broad deficit in inflammatory capacity due to p11 ablation, we evaluated paw edema induced by the TRPV1 agonist capsaicin, which is also present in the same, small DRG neuronal population as PAR2 (Fig. 5B). Subcutaneous injection of capsaicin produced equivalent levels of paw swelling in both p11-WT and p11-KO mice (Fig. 5, E and F). This result confirms that the blunted inflammatory response to AC-55541 is likely a specific consequence of impaired PAR2 signal transduction in the absence of p11, rather than a global defect in sensory neuron function or edema formation.

**Fig. 5.**
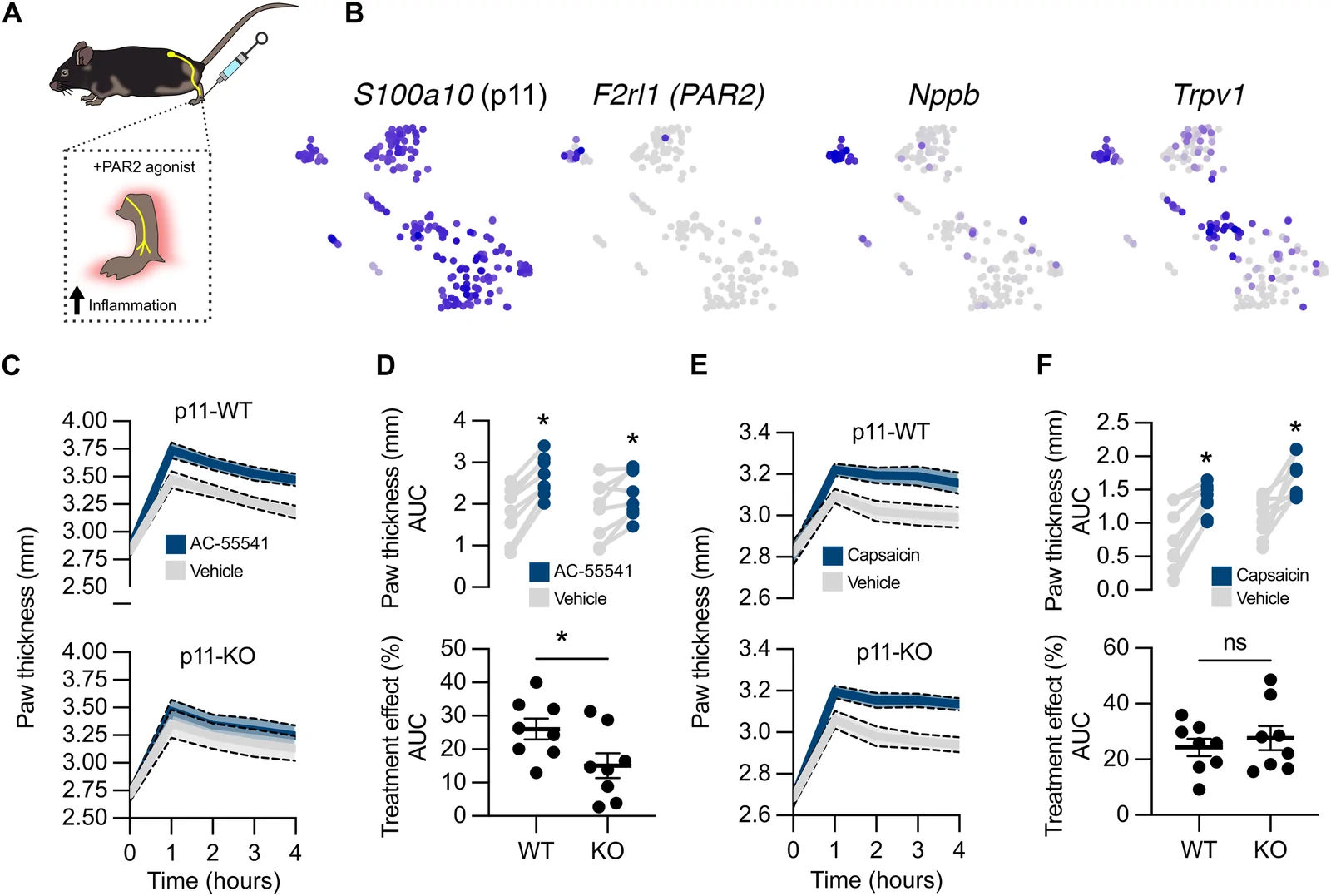
p11 promotes PAR2 agonist–induced edema in vivo. (**A**) Depiction of in vivo experiments to evaluate PAR2 agonist–mediated hind paw inflammation. (**B**) t-SNE analysis of mouse DRG neuron transcriptomes, sourced from Li *et al.* (*33*), identifies that PAR2 (*F2rl1*) is restricted to a specific subset of sensory itch neurons which also express p11 (*S100a10*). This population is also characterized by the coexpression of *Nppb* and *Trpv1*. (**C**) Paw thickness (in millimeters) at baseline and after injection with AC-55541 or vehicle, in p11-WT (top) and p11-KO mice (bottom) (*n* = 8 for both genotypes). (**D**) Top: area under the curve (AUC) of vehicle or AC-55541 injection–induced paw swelling (*P < 0.05 vehicle versus AC-55541, two-way ANOVA with uncorrected Fishers LSD). Bottom: AUC of AC-55541 specific inflammatory effect (in percentage) in both genotypes (*P < 0.05 WT versus KO, unpaired *t* test with Welch’s correction). (**E**) Paw thickness (in millimeters) at baseline and after injection with capsaicin or vehicle, in p11-WT (top) and p11-KO mice (bottom) (*n* = 8 for both genotypes). (**F**) Top: AUC of vehicle or capsaicin injection–induced paw swelling (*P < 0.05 vehicle versus capsaicin, two-way ANOVA with uncorrected Fishers LSD). Bottom: AUC of capsaicin-specific inflammatory effect (in percentage) in both genotypes.

## DISCUSSION

This study characterizes GPCR-p11 interactions by moving from a mechanistic understanding of p11 recruitment to the systematic mapping of its interactome and its functional role as a GPCR signal modulator. We first identified that the p11-interacting receptor 5-HT_4_R, but not the 5-HT_1A_R, recruits p11 to the SPM. Our observation that the TM5/6 helical bundle is the primary determinant of p11 SPM recruitment is consistent with recent structural data defining the G protein interface of the 5-HT_4_R that determines G protein subtype coupling specificity (*24*). Cryo–electron microscopy structures of 5-HT_4_R complexed with Gα_s_ or Gα_i_ reveal that these TM helices undergo significant length changes; specifically, the cytosolic end of the TM5 α helix in the Gα_s_-coupled 5-HT_4_R is extended by 12 residues compared to its Gα_i_-coupled counterpart. Therefore, it is possible that either this structural phenomenon, or the subsequent G protein recruitment enabled by it, allows for p11 to engage the receptor, explaining why replacing this entire helical subdomain in our 5-HT_1A/4_ chimeras was required to abolish p11 SPM recruitment. This translocation expands upon the observation that GPCRs can alter the spatial distribution of p11. Specifically, prior work on mGluR5 demonstrated that p11 can be recruited to regulate receptor localization and depression-like behaviors (*12*). Translocation of p11 to the SPM has also been observed in forskolin- (*34*), histamine- (*35*), and thrombin- (*36*) treated cells. Our real-time ebBRET and endogenous p11 imaging findings build on these observations by demonstrating that active GPCRs can promote p11 surface membrane recruitment across a broader range of receptors in the GPCR superfamily.

Likewise, we show that an active receptor is a primary driver of this physical interaction; the inactive D66N mutant exhibited a significant loss of p11-mVenus recruitment compared to the WT 5-HT_4_R. In addition, p11-mVenus recruitment to the WT 5-HT_4_R was inhibited using an inverse agonist, GR125487. Similarly, the 5-HT_4_R H222R mutant also showed impaired p11-mVenus recruitment relative to WT 5-HT_4_R but remained significantly higher than that of the 5-HT_1A_R control. This result suggests that H222R may sample active conformations less effectively than the WT receptor but retains sufficient structural compatibility for p11 engagement. Alternatively, these data imply that p11 may compete for some of the same residues required for Gα_s_ protein recruitment or that the structural requirements for p11 binding partially overlap with the G protein coupling interface. These experimental observations are conceptually supported by our AF3 models. Consensus models predict that most intermolecular contact residues reside within the cytosolic tail of TM5, a region critical for G protein docking. Furthermore, recent independent proximity-labeling studies investigating the interactomes of two distinct GPCRs have reported p11 recruitment in response to agonist treatment, suggesting that an active conformation may be a widespread prerequisite for p11 engagement across the GPCRome (*37*, *38*). While p11 is the best-described member of the S100 protein family to interact with GPCRs, other members such as S100B function through calcium-dependent structural transitions to modulate downstream pathways of GPCRs such as the 5-HT_7_R or D_2_R (*39*, *40*). Future studies aimed at dissecting the dependence of these interactions of receptor activity would be informative.

Beyond physical recruitment, we observed that receptor activation is associated with a marked increase in cellular p11-mVenus abundance in our cotransfection system. Our data suggest that this phenomenon may be influenced by multiple factors within the experimental system. On one hand, RNA-seq data confirmed that S100A10 mRNA levels increased significantly following receptor stimulation only in p11-mVenus–transfected samples, indicating that signaling-driven stimulation of the CMV promoter likely contributes to the observed p11-mVenus protein pool (fig. S19). On the other hand, the degree of p11-mVenus accumulation following TAAR1 stimulation was notably higher than that of three alternative mVenus-tagged control constructs (RAMP1-3) expressed in the same backbone, suggesting that protein-specific effects may also be at play. Notably, we also observed that coexpression with ACKR3—a receptor that typically does not couple to G proteins in HEK293T cells—was sufficient to drive a significant increase in p11-mVenus abundance (*41*, *42*).

It is well established that p11 is inherently unstable as a monomer and is typically targeted for rapid ubiquitin-mediated proteasomal degradation unless protected by a partner such as ANXA2, which masks its C-terminal lysine residues (*21*, *22*). One possibility is that engagement with an active GPCR provides a similar protective scaffold that shields p11 from the proteasomal machinery, either in the absence or presence of ANXA2. The observation that the 5-HT_4_R-H222R mutant exhibits both impaired p11-mVenus recruitment and a lack of p11-mVenus accumulation suggests that high-affinity engagement may be a prerequisite. This observation is further supported by the substantial reduction in endogenous p11 protein levels we observed in our ANXA2-KO cells (fig. S2D), which highlights the dependence of p11 on physical partners to maintain its intracellular protein levels. Similarly, cotransfection of ANXA2 with p11-mVenus led to a significant increase in p11-mVenus protein levels that correlated with the level of ANXA2 expression (fig. S14A). In addition, cotransfecting 5-HT4R-NLuc and p11-mVenus resulted in overall lower p11-mVenus levels in ANXA2-KO cells than in p11-KO cells, indicating that most of the synthesized p11-mVenus is likely coupled to ANXA2 (fig. S10). Furthermore, as cAMP has been reported to promote p11 association with other partners like CFTR, ANXA2, and TRPV6, signaling-mediated shifts in p11 turnover remain a relevant consideration (*21*, *43*, *44*).

Ultimately, it remains unclear whether the increase in p11 abundance stems primarily from signaling-dependent transgene expression, receptor-mediated stabilization, or a combination of both, as there is evidence for each mechanism. These findings highlight the importance of accounting for signaling-driven protein fluctuations when investigating protein-protein interactions. Since the activity of expression systems can be influenced by GPCR signaling, surges in transgene expression may be inadvertently interpreted as evidence of physical interaction. To address this issue, we used different noninteracting control constructs (mainly the 5-HT_1A_R) alongside systematic physical protein-protein interaction mapping to help distinguish specific engagements from broader fluctuations in protein levels.

We then carried out a multiplexed proteomic screen using an SBA immuno-assay to identify complexes between p11 and 211 heterologously expressed, solubilized human GPCRs. This screen allowed the first large-scale elucidation of the GPCR-p11 interactome and the identification of distinct GPCR-p11 complexes. As a result, we provide evidence for almost two dozen new potential GPCRs that interact with p11. While our screen successfully identified several known interactors, such as 5-HT_4_R, 5-HT_1D_R, and FSHR, it did not confirm earlier reported GPCR-p11 complexes such mGluR5 and 5-HT_1B_R. Several factors likely contributed to this outcome. First, 5-HT_1B_R has been reported as a weaker interactor compared to 5-HT_4_R (*10*), and our stringent hit selection threshold based on the 5-HT_4_R may have led to the exclusion of lower-affinity interactors. Second, receptors like mGluR5 have been reported to engage p11 via their distal carboxyl tails, whereas our screen may favor interactions involving the core helical bundle (*12*). Because the screen was performed on solubilized membranes from unstimulated cells, our data are likely biased toward receptors that exhibit sufficient constitutive basal activity to drive p11 engagement under the specific experimental conditions of the assay. Last, we also recognize the limitation of our solubilization approach; follow-up investigations to resolve GPCR-p11 interactions may also benefit from alternative solubilization or stabilization methods, as active receptor stability may differ for GPCRs across different solubilization methods. Similarly, our screen may also underrepresent receptors that require ligand-induced conformational changes to recruit p11. 5-HT_2B_ emerged as a double hit in our SBA screen. Combined with recent reports that 5-HT_2A_ is an activity-dependent interactor of p11 (*37*), this suggests that p11 may interact with a broader range of the 5-HT receptor family than previously recognized.

Last, we investigated the physiological relevance of the p11 interaction by focusing on PAR2, a high-confidence interactor identified in our screen. In vitro transcriptomic fingerprinting revealed that the p11 presence was associated with both an accelerated and increased endogenous PAR2 transcriptional response. Consistent with these findings, we observed enhanced signals across Gα_q_, Gα_i_, and Gα_12/13_ pathway reporters in cells expressing p11, suggesting that it may function as an endogenous modulator of PAR2 signaling. While previous studies demonstrated that p11 promotes GPCR surface expression, our results indicate that p11 also increases the abundance of cell surface PAR2 following agonist treatment. Several mechanisms could account for this phenomenon, such as p11-mediated deceleration of endosomal internalization rates or structural stabilization of the receptor on the SPM, both of which warrant future investigation. Our findings are consistent with the characteristically slow rate of PAR2 internalization typically induced by AC-55541 (*29*). However, these internalization kinetics may diverge in the presence or absence of p11 when evaluating alternative compounds. Ultimately, our combined data suggest that p11 functions as a signal amplifier for PAR2.

To establish an anatomical basis for this interaction in vivo, we analyzed mouse scRNA-seq data, which indicated that p11 and PAR2 are coexpressed in a subset of sensory neurons within the DRG. Guided by this coexpression pattern, we used a global p11-KO mouse model to evaluate the physiological impact of this relationship. Consistent with our cellular models, p11-deficient mice exhibited a reduced inflammatory response following PAR2 activation, characterized by a decrease in paw edema compared to WT controls. It appears that by modulating PAR2 signaling, p11 may influence the threshold for inflammatory or nociceptive signaling within these specific sensory circuits. By demonstrating that p11-KO mice exhibit an unaltered inflammatory response to the TRPV1 agonist capsaicin, we rule out the possibility that the blunted edema seen with AC-55541 in p11-KO mice is merely a by-product of generalized inflammatory dysfunction. This functional isolation positions the PAR2-p11 interface as a specific modulator of neurogenic inflammation within these circuits. Further investigation into other relevant hits, such as the opioid peptide scavenger receptor ACKR3, would be informative regarding the broader role of p11 in GPCR-mediated nociception. Notably, p11 has been previously implicated in the surface trafficking of two nociceptive ion channels expressed in the DRG, Na_v_1.8 and ASIC1a, and mice with p11 ablated in primary sensory neurons show an attenuation of acute and neuropathic pain (*45*–*47*). Collectively, these results indicate that p11 is a regulator of nociception within the DRG which may involve multiple GPCRs.

In summary, our results show that p11 is an activity-dependent modulator across the GPCRome. By influencing GPCR-mediated signaling, p11 appears to regulate the sensitivity of physiological circuits, such as those found in sensory neurons, suggesting that the GPCR-p11 interface is an attractive avenue for future therapeutic study.

## MATERIALS AND METHODS

### Cell culture maintenance and transfections

HEK293T (obtained from American Type Culture Collection, CRL-3216), p11-KO, and ANXA2-KO cells were maintained in Dulbecco’s modified Eagle’s medium (DMEM) supplemented with 10% fetal bovine serum (FBS), GlutaMAX, sodium pyruvate, Hepes, nonessential amino acids, and penicillin-streptomycin (PS) (Gibco). Cells were cultured in 100-mm cell culture dishes (Sarstedt) at 5% CO_2_, 37°C, and 95% humidity. Expi293F cells were maintained in 8% CO_2_, 37°C, and 95% humidity and 125-ml shaking flasks (Corning) using Gibco Expi293 Expression Medium.

All transfections in experiments involving cells other than Expi293F were conducted using polyethyleneimine (PEI) (Transporter 5, Polysciences) at a DNA to PEI ratio of 1:4, unless otherwise specified. When necessary, empty pcDNA3.1(+) plasmid was added to maintain a consistent gene dose of 2 pg total DNA per cell.

### HEK293T KO cell line generation

Design, editing, and single-cell sorting of *S100A10* (p11)-KO and *ANXA2*-KO cell lines were performed by the CRISPR Functional Genomics unit at Karolinska Institutet. Briefly, two single guide RNAs (sgRNAs) targeting each gene locus were used (p11-KO: 5′-CAAATGGAACACGCCATGG-3′ and 5′-CACTTACTTCCAAAAATCCA-3′; ANXA2-KO: 5′-CTCAGCATCAAAGTTAGTAT-3′ and 5′-ACAGACCCATATGCACTTGG-3′) (CRISPRevolution sgRNA EZ Kit, Synthego). sgRNAs (1000 ng) and Cas9 protein (5000 ng; Alt-R *S.p.* Cas9 Nuclease V3, IDT) were precomplexed into ribonucleoproteins and introduced into cells via electroporation (Neon System, Thermo Fisher Scientific; 1500 V, 30 ms, 1 pulse). Allelic disruption frequency was estimated by droplet digital polymerase chain reaction (PCR) (QX200, Bio-Rad). HEK293T cells were single-cell sorted (SONY SH800) into 96-well plates to generate monoclonal lines. Clones were expanded and validated for genomic deletions via PCR and Sanger sequencing. Protein KO was confirmed by Western blot (ANXA2) or flow cytometry (p11).

### Membrane solubilization

Cell membranes were solubilized using a method described previously (*20*). Cells expressing GPCRs and/or p11 were incubated in a solubilization buffer [50 mM Hepes, 1 mM EDTA, 150 mM NaCl, and 5 mM MgCl_2_ at pH 7.4, containing 1% (w/v) *n*-dodecyl β-d-maltoside (DM) and cOmplete mini protease inhibitor] for 2 hours at 4°C with gentle agitation. After solubilization, the lysates were clarified by centrifugation at 22,000*g* for 20 min at 4°C. The resulting solubilized lysates were transferred to a microcentrifuge tube, and the total protein content was determined using a BCA assay following the manufacturer’s guidelines. Last, the solubilized lysates were promptly flash-frozen for storage.

### Plasmid construct generation

DuET GPCR library generation is described elsewhere (*20*). For generating NLuc tagged GPCR constructs, DuET GPCR backbone was amplified without the C-terminal 1D4 tag and was replaced with a NLuc tag using Gibson assembly cloning (Gibson Assembly Kit, NEB). CalLuc 2.1, HiBiT-PAR2-mScarlet3, p11-OLLAS, and rGFP-CAAX were synthesized and generated by GenScript. Gibson assembly was also used to generate p11-mVenus and p11-RLucII, for which the human p11 was amplified from a p11-FLAG eGFP-tagged construct obtained from GenScript. 5-HT_1A_R–mScarlet was generated using Gibson assembly by replacing the 1D4-epitope tag from the DuET 5-HT1A construct with mScarlet from an existing in-house DRD2-mScarlet construct. ANXA2-FLAG was also obtained from GenScript. 5-HT_4_R mutants were generated using Gibson assembly cloning (5-HT_4_R ICL3 short: S235-T248 was replaced with E234-R323 from 5-HT_1A_R; 5-HT_4_R ICL3 long: 5.67 to 6.30 was replaced with E234-R323 from 5-HT_1A_R; 5-HT_4_R 5.71 to 6.30: 5.71 to 6.30 was replaced with 5.71 to 6.30 from 5-HT_1A_R; 5-HT_4_R TM5 and TM6: 5.35 to 6.61 was replaced with 5.35 to 6.61 from 5-HT_1A_R). All constructs were verified by Sanger sequencing.

### Immunoprecipitation and Western blotting

Clarified membrane lysates were incubated with an anti-1D4 mAb (in-house) or IgG isotype control (Thermo Fisher Scientific, 31903) overnight before incubation with anti-protein A/G magnetic beads (Thermo Fisher Scientific, 88802) according to the manufacturer’s instructions. Reduced elutants containing GPCR-p11 complexes were run on a 4 to 12% bis-tris SDS gel (Invitrogen), transferred to polyvinylidene difluoride membrane (Bio-Rad), and blotted with an anti-p11 mAb (1:100, BD Biosciences, 610071) diluted in Intercept TBS blocking buffer (LI-COR).

### Membrane preparation

Expi293F cells were cultured and transfected following the manufacturer’s instructions. For transfection, the cells were diluted to a concentration of 2 × 10^6^ cells/ml and allowed to grow overnight. The next day, the cells were diluted to 3 × 10^6^ cells/ml, and 1.25 ml of the cell suspension was transferred to each well of a 12-well culture plate. Transient transfections were then performed using the ExpiFectamine 293 transfection kit (Thermo Fisher Scientific) according to the manufacturer’s instructions. Each well was transfected with 4 μl of ExpiFectamine Reagent, 0.25 μg of GPCR plasmid, and 0.25 μg of p11-OLLAS plasmid, maintaining a total plasmid DNA amount of 1.5 μg per well with the addition of empty pcDNA3.1(+). Enhancers were added 18 to 24 hours posttransfection, and the cells were harvested 72 hours posttransfection. For control purposes, untransfected Expi293F cells were grown for 48 hours in 12-well plates (Sarstedt) at the same density (3 × 10^6^/ml) as the transfected cells. To harvest the cells, each well was collected to a 1.5-ml Eppendorf tube, spun down at 4°C, and washed twice with ice-cold phosphate-buffered saline (PBS). The pellets then underwent membrane solubilization described earlier to generate the material for the SBA screen.

### SBA generation

Anti-p11 and anti-tag Abs were covalently attached to color-coded beads (MagPlex, Luminex Corp.). Each Ab was affixed to a specific bead identification population, as described previously. A total of 1.75 μg of each Ab was mixed in MES buffer [100 mM 2-(*N*-morpholino)ethanesulfonic acid (pH 5.0)] to achieve a final volume of 100 μl. The magnetic beads were then activated using *N*-hydroxysuccinimide (Pierce) and 1-ethyl-3-(3-dimethylaminopropyl)-carbodiimide (ProteoChem). Following 20 min of activation, the diluted Abs were linked to the activated carboxylated beads using NHS-EDC chemistry. After a 2-hour incubation period in darkness with nutation, unbound Abs were removed by washing with PBS containing 0.05% Tween 20 (PBS-T) before adding BRE buffer (Roche) supplemented with ProClin 300 (Sigma-Aldrich) and incubated overnight. On the subsequent day, Ab-bound beads were organized into groups and pooled to create the SBAs. The coupling efficiency was evaluated using anti–rabbit-RPE and anti–mouse-RPE Abs (Jackson ImmunoResearch), as most Abs linked to the beads were rabbit polyclonal Abs or mouse mAbs. Data acquisition was performed using a FlexMap 3D instrument (Luminex Corp.) with xPONENT Software, version 4.3.309.1.

### SBA assay

Clarified protein lysates were diluted to a concentration of 2 μg/μl in solubilization buffer (as previously described) with 0.01% (w/v) DM. These were placed in a 96-well unskirted PCR plate and further diluted 3.6 times in SBA assay buffer. For each assay, 12.5 μl of the lysate (equivalent to 25 μg) was mixed with 32.5 μl of assay buffer, achieving a final volume of 45 μl. This mixture was then transferred to a 384-well assay plate, with each well already containing 5 μl of bead array, using a CyBio SELMA liquid handling system (Analytik Jena). For the readout, lysates and beads were incubated overnight for 16 hours at 4°C. The plates were then washed six times with 60 μl of PBS-T using a BioTek EL406 washer. Subsequently, 50 μl of phycoerythrin (PE)–conjugated anti-tag detection Abs diluted in BRE containing 0.1% DM, 0.1% Tween 20, and 10% rabbit IgG was added to each well, and the plates were incubated for 1 hour at 4°C. The following dilutions were used: PE-conjugated anti-1D4, 1:1000; PE-conjugated anti-OLLAS, 1:500. PE-conjugated anti-1D4 and PE-conjugated anti-OLLAS were generated using the anti-1D4 and anti-OLLAS Abs available in-house and a commercial PE conjugation kit (Abcam).

Following the incubation period, the beads underwent three wash cycles with six wash cycles using 60 μl of PBS-T. Next, 60 μl of PBS-T was added to the beads. The fluorescence signal associated with each bead was then assessed using a Luminex FlexMap 3D instrument (Luminex Corp.) operated with xPONENT Software, version 4.3.309.1.

### SBA data analysis

To normalize measurements from different antibodies, raw MFI values were transformed into robust *z*-scores for each capture-detection scheme using the formula: [*x* − median(*x*)]/[1.4826 ∗ MAD(*x*)], where *x* represents the measurement values from a single Ab, and MAD denotes the median absolute deviation. Interactions were identified by calculating cutoffs for each capture-detection scheme. This was done by adding 6 MADs (with a constant of 1.4826) to the median of samples containing only GPCR transfection. GPCR-p11 samples with a robust *z*-score above this cutoff were considered to interact.

### GPCR-p11 BRET assays

HEK293T WT, p11-KO, or ANXA2-KO cells were transfected and seeded in universal signaling assay media (USAM) (FluoroBrite DMEM, 5% dialyzed FBS, and 1% PS). Cells were seeded into 96-well plates (Corning) at a concentration of 50,000 cells per well and 90 μl of media. The transfection gene dose is specified in the figure legend for each experiment. Baseline BRET measurements were performed 24 hours posttransfection, and endpoint overnight ligand stimulation reads were performed 44 hours posttransfection, with a ligand stimulation time of 20 hours. mVenus fluorescence was measured using a Tecan SPARK microplate reader (excitation: 485/20 nm, emission: 535/25 nm) before the addition of 10 μM coelenterazine 400a (NanoLight). Total luminescence was read for 5 min to ensure signal stability before multicolor luminescence readings to determine BRET. NLuc luminescence was recorded between 360 and 440 nm, and acceptor emission (mVenus) was detected between 505 and 570 nm. Both light emissions were recorded with an integration time of 100 ms. Kinetic GPCR-p11 assays were performed in identical conditions with a few modifications; Vivazine (1:1000 dilution, Promega) was added 1 hour before the initial BRET measurements, and plates were read every 10 min in 5% CO_2_, 37°C, and in the presence of a humidity cassette.

### p11 membrane recruitment assay

For p11 membrane recruitment, ANXA2-KO cells were transfected and seeded in growth media (DMEM, 10% FBS, and 1% PS) into white PDL-coated 96-well plates (Greiner) at a concentration of 50,000 cells per well and 100 μl of media. A total of 2 pg of DNA was used per cell (10% p11-RLuc, 40% rGFP-CAAX, 5% GPCR or 10% ANXA2, and pcDNA3.1+ were added to a total amount of 2 pg DNA per cell) with a 2:1 PEI:DNA ratio. Four hours before BRET reading, media was changed to starvation media (DMEM and 1% PS). For BRET reading, cells were washed once with Hanks’ balanced salt solution (HBSS) without phenol red (Gibco) and kept in 90 μl of HBSS. Donor (RLucII) luminescence was recorded between 360 and 440 nm, and acceptor emission (rGFP) was recorded between 505 and 570 nm at an integration time of 100 ms, 5 min after 10 μl of coelenterazine 400a was added.

### cAMP measurements

GloSensor 22-F cAMP sensor (Promega) was transfected along with receptor/p11/pcDNA to Expi293F cells (3 × 10^6^ cells/ml) using the gene doses specified in the figure legends. The transfection was performed using ExpiFectamine at a DNA to ExpiFectamine ratio of 1:3.2 in 2 ml of media, and the cells were maintained in six-well plates. Forty-eight hours after transfection, the cells were spun down and resuspended in assay buffer [HBSS, 10 mM Hepes, 2 mM d-luciferin (Promega), and 300 μM 3-isobutyl-1-methylxanthine (Sigma-Aldrich)] and seeded with 25,000 cells per well to a white 384-well plate (CulturPlate, PerkinElmer) and incubated for 1 hour in the dark at room temperature (RT). The plate was read three to five times to establish a baseline signal before compound addition, after which it was read every 10 min for 1 hour. The last measurement was used for calculating cAMP responses.

### PRESTO-TANGO assay

HTLA cells (provided by B. Roth, UNC School of Medicine) were transfected in USAM using gene doses of the PAR2-TANGO plasmid (Addgene plasmid #66277) specified in the figure legends. The total DNA amount was balanced to 100 ng per well using an empty vector. The cell-DNA mixture was seeded into white 96-well plates (Costar) at a density of 50,000 cells per well. The cells were stimulated 24 hours posttransfection with AC-55541 for 16 hours. Following stimulation, Bright-Glo reagent was added to each well at a 1:10 volume ratio, and luminescence was measured using a TECAN Spark microplate reader.

### PAR2 cell surface expression assay

p11-KO cells were transfected in USAM with 0.1 ng of HiBiT-PAR2-mScarlet3 combined with either 50 ng of p11-mVenus plus 50 ng of empty pcDNA vector or 100 ng of empty pcDNA vector alone (control). The cell-DNA mixture was seeded into a white 96-well plate at a density of 50,000 cells per well in a total volume of 90 μl. After 24 hours, the cells were treated with 10 μM AC-55541 or a vehicle control. Following stimulation, 50 μl of Nano-Glo HiBiT Extracellular Detection Reagent was added to each well. The plates were incubated at room temperature for 2 min, and luminescence was subsequently recorded for 5 min using a TECAN Spark microplate reader.

### Endogenous PAR2 signaling measurements

p11-KO cells were transfected with either pGL4.29[*luc2P*/CRE/Hygro], pGL4.30[*luc2P*/NFAT-RE/Hygro], pGL4.33[*luc2P*/SRE/Hygro] or pGL4.34[*luc2P*/SRF-RE/Hygro] (Promega), and p11-mVenus plasmids at the gene doses stated in the figure legends. Cells were seeded and transfected in USAM at a concentration of 50,000 cells per well. Twenty-four hours after transfection, cells were stimulated with AC-55541 and incubated for a further 5 hours. BrightGlo (1:10, Promega) was added to each well, and total luminescence was read three times.

To measure calcium signaling, p11-KO cells were transfected in suspension. Transfection complexes containing a total of 100 ng of plasmid DNA per well [50 ng of the bioluminescent calcium sensor CalLuc 2.1 (*48*) and 50 ng of p11-mVenus or empty pcDNA vector] were mixed directly with the cell suspension before seeding into assay plates in USAM at a concentration of 50,000 cells per well. The following day, a 10× substrate spike-in master mix was prepared by combining 100 mM d-luciferin and 100 mM Hepes stocks in a 1:4 ratio. A 10-μl volume of this mix was added to each well, and the plates were incubated for 2 hours at room temperature. Baseline luminescence was recorded for 5 min, followed immediately by test compound addition and continuous plate reading for an additional 10 min.

### Live-cell imaging assay

For live-cell imaging experiments, 6.0 × 10^5^ S100A10-OpenCell HEK293T cells were suspended in 1.8 ml of USAM and seeded into Ibidi 35-mm μ-dishes. Transient transfections were performed using a DNA to PEI ratio of approximately 1:4, with a total gene dose of ∼1.83 pg total DNA per cell. Transfection complexes were prepared by combining either 0.55 pg per cell of HiBiT-PAR2-mScarlet3 or 0.55 pg per cell of 5-HT_1A_R–mScarlet with 1.28 pg per cell of empty pcDNA3.1(+) vector to maintain a consistent total DNA amount. Twenty-four hours posttransfection, cells were treated with or without their respective agonists before imaging. Confocal microscopy was performed on a Zeiss LSM 880 microscope using a 40× objective lens equipped with lasers and detectors configured for mScarlet, brightfield, and mNeonGreen2 channels. Z-stacks encompassing the cells in focus were acquired. Downstream image processing and analysis were carried out using QuPath software. For quantification, an optical slice corresponding to the approximate center plane of the cell cluster was selected. Cell boundaries were annotated manually using structural features visible across all three imaging channels. These compiled regions of interest were shrunk to define a ring-like mask isolating the cell SPM. Last, the MFI of the green channel (endogenous p11-mNeonGreen2) was quantified within the ring mask of successfully transfected cells.

### SBA hit screening with a plate reader

p11-KO cells were seeded and transfected in suspension using USAM at the indicated gene ratios (1:1 or 1:5 of DuET GPCR to p11-mVenus). Cells were seeded in black, clear-bottom 96-well plates (Greiner) at a density of 50,000 cells per well and incubated for 24 hours at 5% CO_2_, 37°C, and 95% humidity. The next day, the plate was read using a Tecan SPARK microplate reader with cell confluency measurements before mVenus fluorescence measurements. The fluorescence intensity values were normalized to the confluency values and then used in subsequent analyses.

### Flow cytometry

For measuring 5-HT_4_R chimera construct surface expression, ANXA2-KO cells were transfected in six-well plates with the indicated constructs (2 pg DNA per cell) for 24 hours. Following transfection, cells were collected by scraping into Eppendorf tubes, spun down, and incubated in flow buffer (PBS + 2% FBS) containing live-death dye (1:1000, 65-0863-14 Thermo Fisher Scientific) for 10 min on ice. Cells were washed and then fixed (Cytofix, BD). Next, cells were washed and incubated with APC-conjugated anti-FLAG mAbs (1:200, ab72569, Abcam) on ice for 30 min. Cells were washed before data acquisition and analysis using a desktop flow cytometry station (Guavacyte, Millipore).

To validate the SBA hits, a DNA preplate with all double positive DuET GPCR hits along with the negative control 5-HT_1A_R was prepared to facilitate transfection ease. A 1:1 gene dose (1 pg receptor DNA and 1 pg p11-mVenus DNA per cell) was transfected to p11-KO cells in suspension, and three technical replicates were seeded into clear 96-well plates (Sarstedt). Twenty-four hours later, media was removed, and all wells were incubated with 50 μl of TrypLE enzyme (Gibco) for 5 min at RT. Cells were detached and transferred to a new V-bottom 96-well plate (Sarstedt) and spun down. TrypLE enzyme was removed, and cells were resuspended in 100 μl of flow buffer containing live-death dye, on ice, for 10 min. The plate was then spun down, and cells were resuspended in 100 μl of cytofix for 20 min on ice. Next, the wells were washed two times with 50 μl of cytoperm (BD) and then incubated for 1 hour on ice with 50 μl of flow buffer containing anti-FLAG APC-conjugated mAbs. After incubation, the cells were washed twice before data acquisition. Data were acquired using an LSR Fortessa Flow Cytometer (BD) equipped with a high-throughput sampler and analyzed with FlowJo software v.10 (BD).

### RNA sequencing

p11-KO cells were seeded into six-well plates and grown to a density of 70% in USAM before transfection. Cells were transfected with either pcDNA (2 pg DNA per cell) or p11-mVenus (1 pg DNA per cell + 1 pg pcDNA DNA per cell) using PEI as a transfection reagent. The next day, transfection efficacy (>70%) was verified by fluorescence microscopy, and cell media was switched to FluoroBrite DMEM without FBS to minimize cell signaling from serum components. AC-55541 (50 μM) was diluted in preheated FluoroBrite DMEM and added to the cells starting from the last time point (8 hours) finishing at 0 hours to ensure that the samples did not differ in cell cycle progression. The media was removed followed by the addition of 400 μl of RLT Buffer (QIAGEN) to instantly lyse the cells. RNA was extracted from the lysate using the QIAGEN RNeasy mini kit (QIAGEN) according to the manufacturer’s instructions. Total RNA was subjected to quality control with Agilent Tapestation according to the manufacturer’s instructions. To construct libraries suitable for Illumina sequencing, the Illumina stranded mRNA prep ligation sample preparation protocol was used with starting concentration between 25 and 1000 ng total RNA. The protocol includes mRNA isolation, cDNA synthesis, ligation of adapters, and amplification of indexed libraries. The yield and quality of the amplified libraries were analyzed using Qubit by Thermo Fisher Scientific, and quality was checked by using Agilent Tapestation. The indexed cDNA libraries were normalized and combined, and the pools were sequenced on the Illumina Novaseq X plus, 10B flowcell, generating 150 base-paired end reads with dual index 10 + 10 base pairs.

Differential expression analysis was performed using the edgeR package in R. To account for library size variations, counts were normalized, and the generalized linear model framework was applied. We estimated tagwise dispersion to account for gene-specific variability across the dataset. *P* values were adjusted for multiple testing using the Benjamini-Hochberg procedure to control the false discovery rate (FDR). Genes were defined as significantly differentially expressed if they had an FDR < 0.05.

### Interaction interface prediction

An ensemble of 100 structural predictions for the 5-HT_4_R (N- and C-terminal sequences removed), and p11 adaptor complex was generated using the AF3 web server and ranked by iPTM scores to select the 20 highest-confidence models for downstream analysis. To systematically identify the binding interface, a custom PyMOL script was used to automate interaction mapping across the selected ensemble. The script iteratively loaded each model, isolated the respective chain identifiers, and applied internal distance selection commands to calculate spatial proximities between the molecular surfaces. Specifically, a 5-HT_4_R amino acid was defined as an interface residue only if it contained at least one heavy atom within a ≤4.0-Å radius of any p11 heavy atom. By tabulating the binary interaction status of each 5-HT_4_R residue per model, the pipeline calculated a consensus interaction frequency, quantifying the percentage of models in which each interchain contact formed to establish a map of predicted intermolecular contacts.

### Single-cell analysis

Single-cell transcriptomic data of mouse DRG neurons was retrieved from the National Center for Biotechnology Information Gene Expression Omnibus under the accession number GSE63576 (*33*). Data processing and visualization were performed using the Seurat package in R. Highly variable features were identified, and the data were scaled and subjected to principal components analysis. The first 10 principal components were used for graph-based nearest-neighbor finding, clustering (resolution = 0.5), and generating t-SNE coordinates. Continuous gene expression gradients for *S100a10*, *F2rl1*, *Nppb*, and *Trpv1* were subsequently mapped onto the t-SNE dimensionality reduction space.

### Animal studies

Male and female WT and p11-KO mice (3 to 4 months old) were used for paw edema assays (WT, *n* = 8; p11-KO, *n* = 8). p11-KO mice were generated as previously described (*9*). Animals were housed under controlled temperature (22°C) and humidity (50%) conditions on a 12-hour light/dark cycle, with ad libitum access to food and water. Tests were performed during the light phase of the cycle. All experiments were approved by the local ethical committee at Karolinska Institutet (N3218-2022) and conducted in accordance with the European Communities Council Directive of 24 November 1986 (86/609/EEC).

### Paw edema assay

Mice were lightly anesthetized with isoflurane (1.3 to 1.5% for maintenance), and dorso-ventral paw thickness was measured as an index of edema using a caliper with 25-μm resolution. The site of subsequent injection was marked with a pen, and baseline measurements were obtained immediately before injection, followed by repeated measurements at 1-hour intervals for up to 6 hours postinjection. Minimal or no compression was applied to the paw during measurements. Separate cohorts of mice received intraplantar injections of either the PAR2 agonist AC55541 (1 μg) or the TRPV1 agonist capsaicin (5 μg) into the left paw, with their respective vehicles [1:2 dimethyl sulfoxide (DMSO):saline and 1:1:8 ethanol:Tween 20:saline, respectively] injected into the contralateral (right) paw. Injections were performed using 10-μl Hamilton syringes fitted with 30-gauge needles, as previously described (*49*–*51*). The skin was punctured proximal to the heel, and the needle tip was advanced subcutaneously toward the central plantar region, where a volume of 10 μl was injected. To maintain hydration during the experiment, animals received an intraperitoneal injection of warmed saline (10 ml/kg) after the 3-hour time point. Paw swelling was quantified as the area under the curve (AUC) of changes in paw thickness relative to baseline over time. Data were analyzed using GraphPad Prism v10.

## Supplementary Materials

### The PDF file includes:

Figs. S1 to S20 Legend for table S1 References
