# Supplementary material for: Systematic identification of p11 as a signaling modulator across the GPCRome
Source: Sci Adv. 2026 Sep 30;12(40):eaeg6567. doi: 10.1126/sciadv.aeg6567 (PMC13626081; doi:10.1126/sciadv.aeg6567)
Supplement: Supplementary file 1 [file sciadv.aeg6567_sm.pdf]

Science Advances
AAAS

Supplementary Materials for

Systematic identification of p11 as a signaling modulator across the GPCRome

Marcus Saarinen *et al.*

Corresponding author: Marcus Saarinen, marcus.saarinen@scilifelab.se; Per Svenningsson, per.svenningsson@ki.se

Sci. Adv. **12**, eaeg6567 (2026)
DOI: 10.1126/sciadv.aeg6567

The PDF file includes:

Figs. S1 to S20
Legend for table S1
References

Other Supplementary Material for this manuscript includes the following:

Table S1

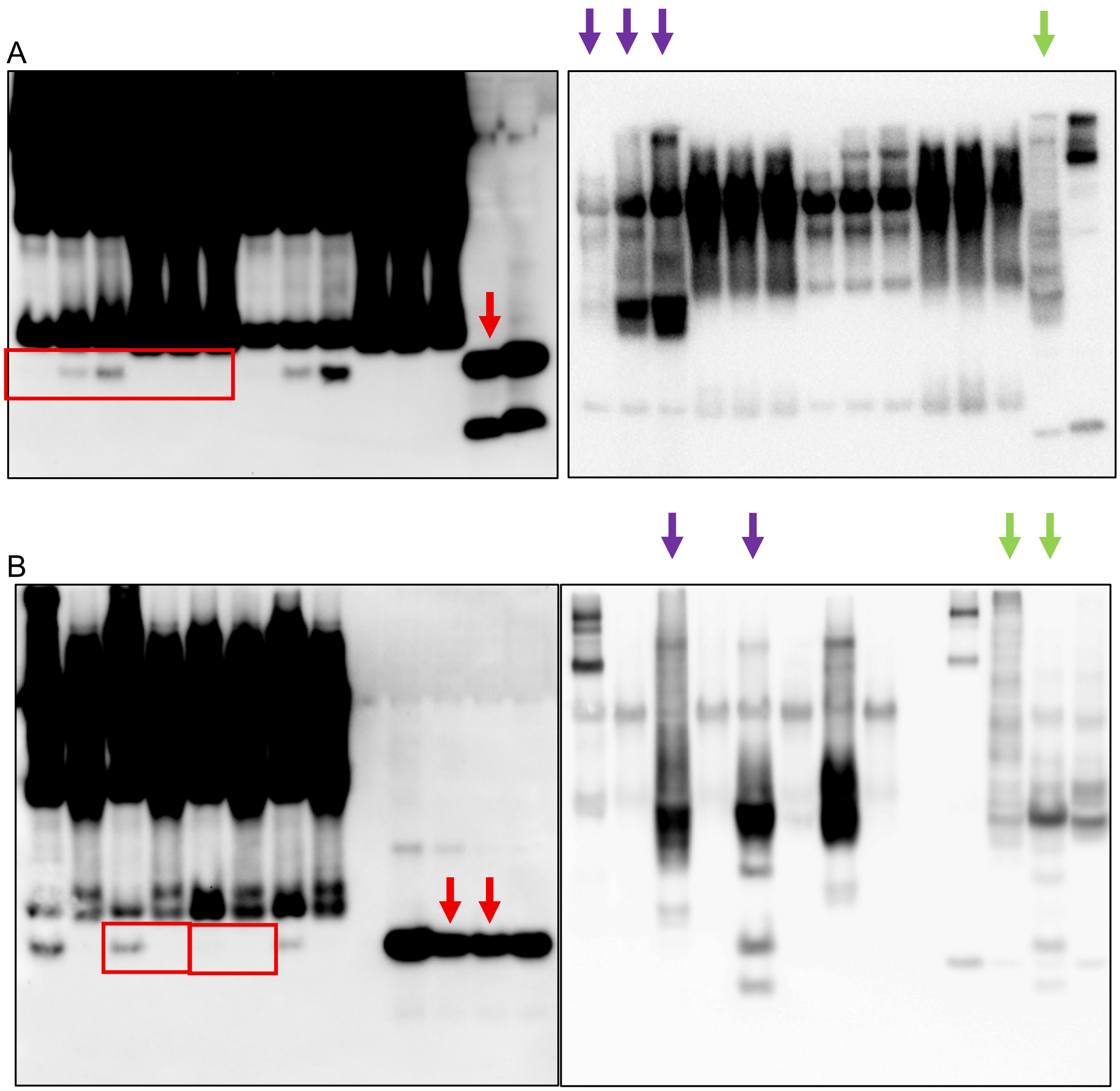

Fig. S1: Uncropped images of gels shown in Figure 1A (A and B). *Left:* Red boxes indicate the selected regions, and red arrows point to p11-OLLAS in the input of the same sample used for the pull-down. *Right:* Membranes are stained with anti-1D4 mAb to quantitate receptor presence on the input (green arrows) or the pull-down (purple arrows).

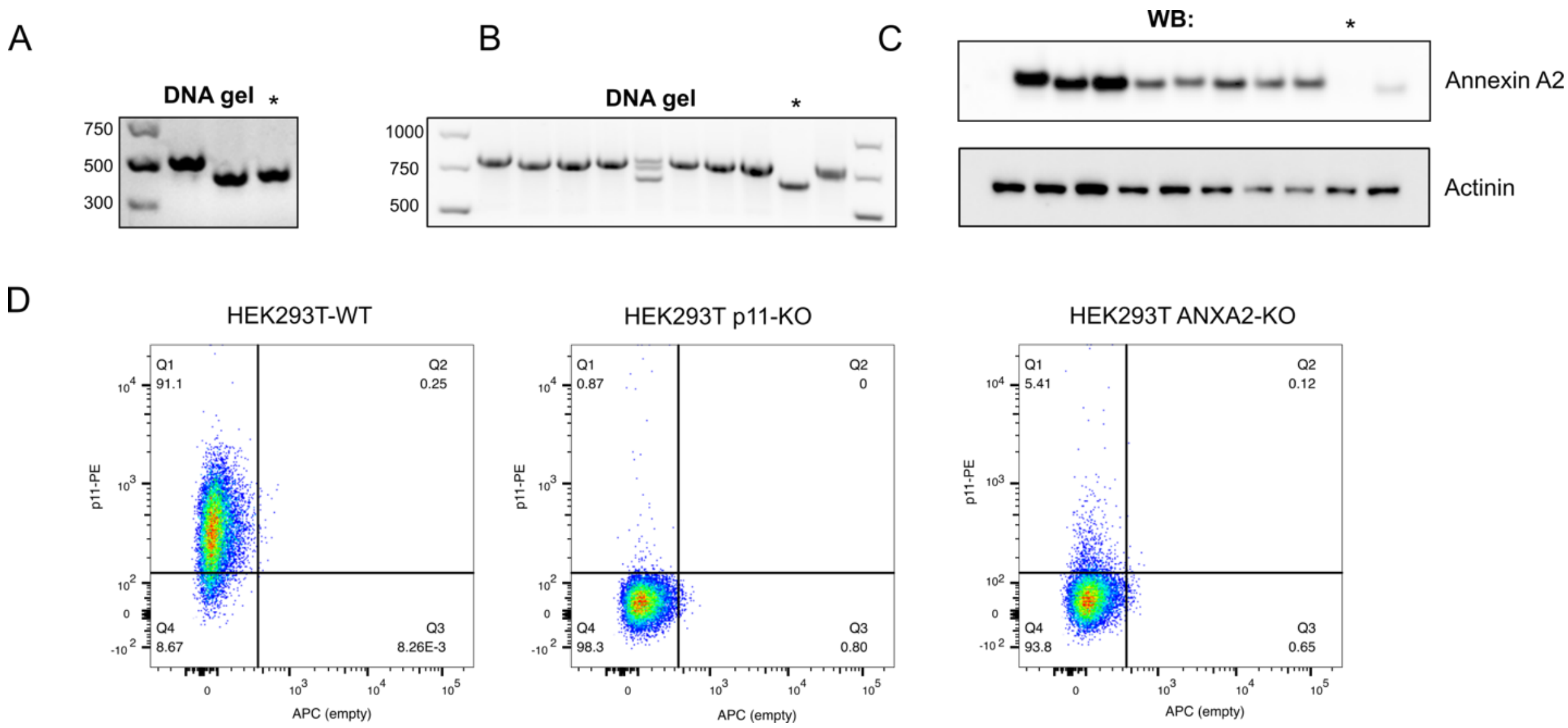

Fig. S2: p11-KO and ANXA2-KO HEK293T generation

(A) DNA gel displaying PCR products of the targeted region amplified from isolated DNA in HEK293T-WT, p11-KO clone 1 and p11-KO clone 2 (with the selected clone for expansion marked with "*") DNA ladder (bp) shown on the left.

(B) DNA gel of from different ANXA2-KO clones, which underwent PCR for the targeted region to be excised. Selected clone for expansion is marked with a "*". DNA ladder (bp) shown on the left.

(C) Western blot to validate ANXA2 protein level knockdown for the chosen clone with actinin as a loading control (bottom panel).

(D) Flow cytometry of HEK293T-WT cells (left panel) and chosen p11-KO clone (middle panel) stained against human p11 using a phycoerythrin (PE) conjugated human p11 Ab (MFI of PE channel on the Y axis) validating global knockdown of p11 protein. A large reduction in p11 levels is also observed in ANXA2-KO cells (right panel).

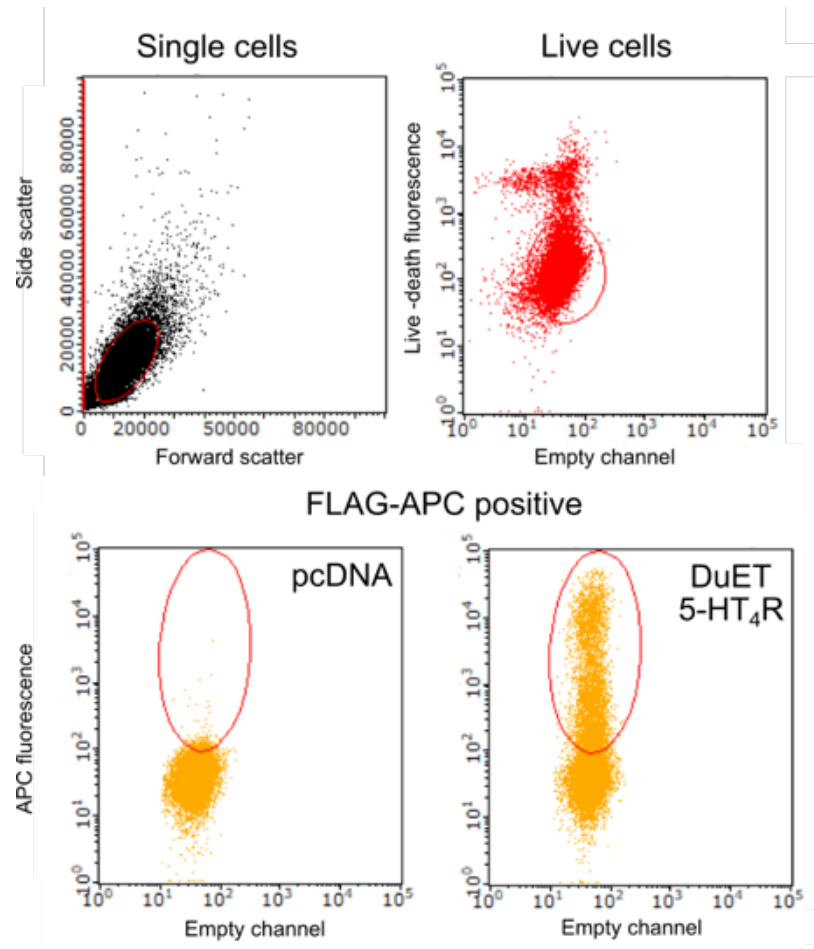

Fig. S3: Surface staining of 5-HT$_4$R receptor constructs

Gating used for selecting single, live and FLAG-APC positive cells, indicated by the red circles.

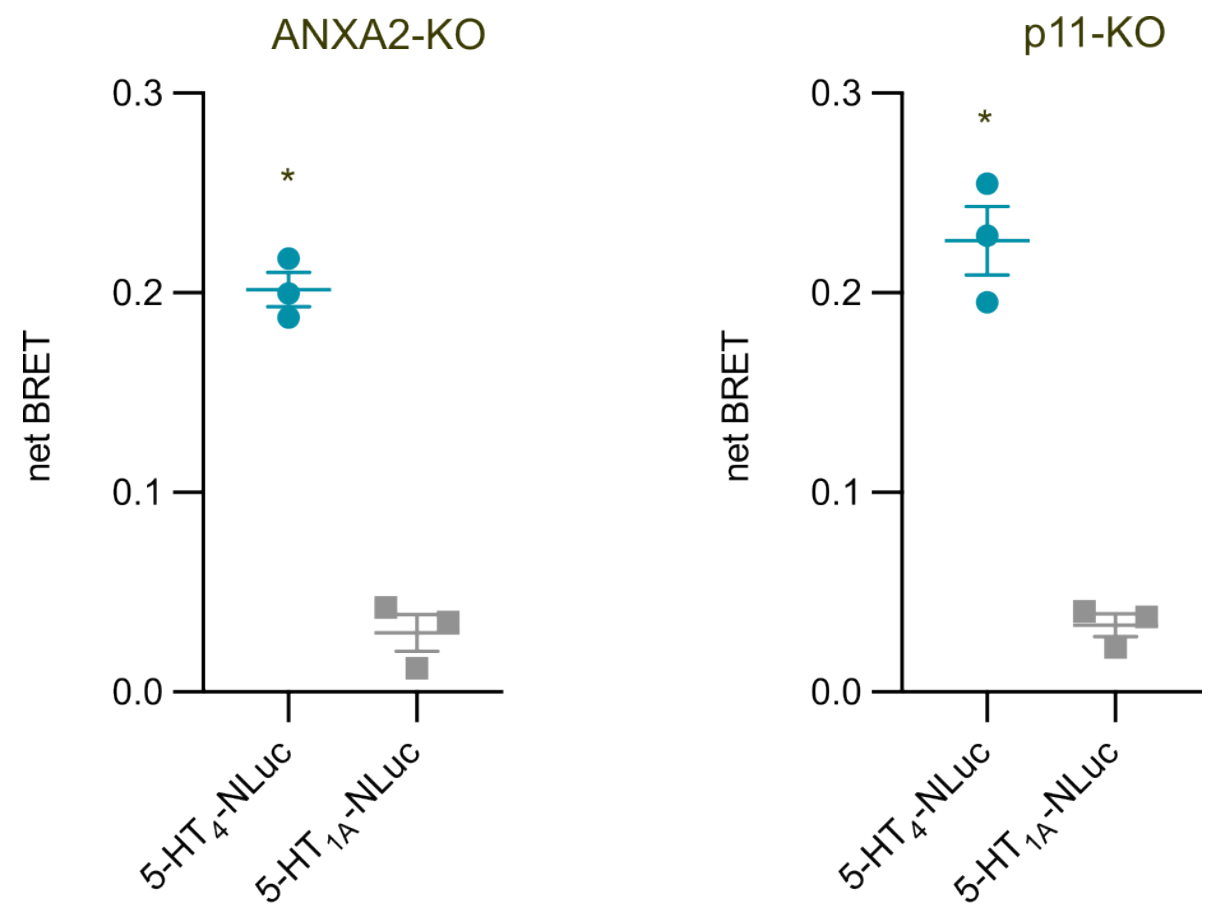

Fig.S4: Validation of BRET assay for p11 interacting GPCRs

netBRET results from 5-HT_4R-NLuc or 5-HT_{1A}R-NLuc and p11-mVenus co-transfected samples in ANXA2-KO or p11-KO (0.002 pg receptor DNA/cell, 1 pg p11-mVenus DNA/cell) (*p<0.05, paired t-test, two -tailed)

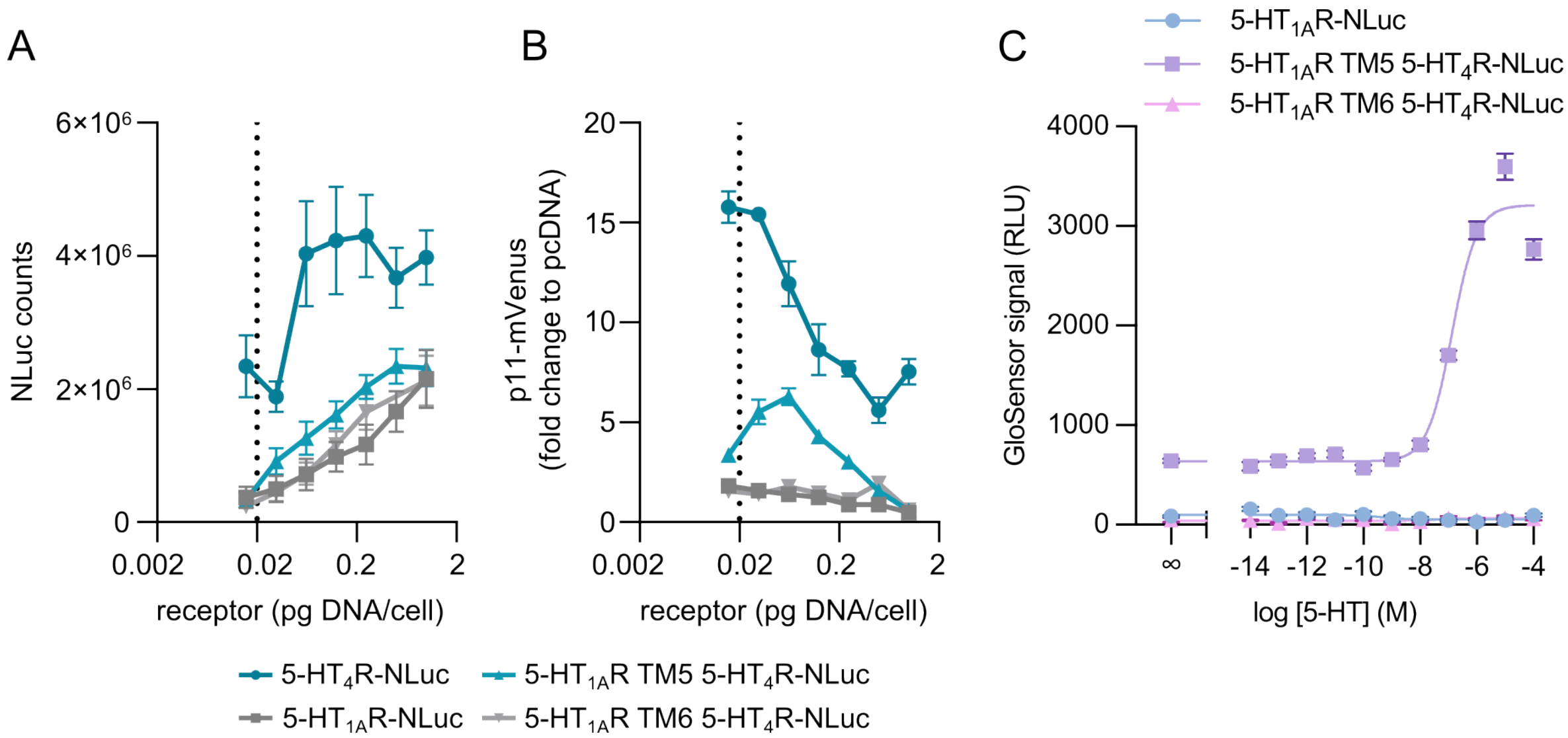

Fig.S5: Further analysis of 5-HT1AR 5-HT4R chimeric constructs

(**A)** Receptor NLuc counts at various gene doses (*n*=4, mean ± SEM). Dotted line indicates receptor gene dose used in Figure 2B.

(B) p11-mVenus fluorescence counts using a constant gene-dose (1 pg DNA/cell) of p11-mVenus with increasing receptor gene dose co-transfection, normalized as a fold-change to co-transfection with empty pcDNA (*n*=4, mean ± SEM)

(**C)** GloSensor cAMP sensor signal when co-expressed with the receptor chimeras or the 5-HT1AR WT and stimulated with 5-HT (*n*=4-6 technical replicates, mean ± SEM)

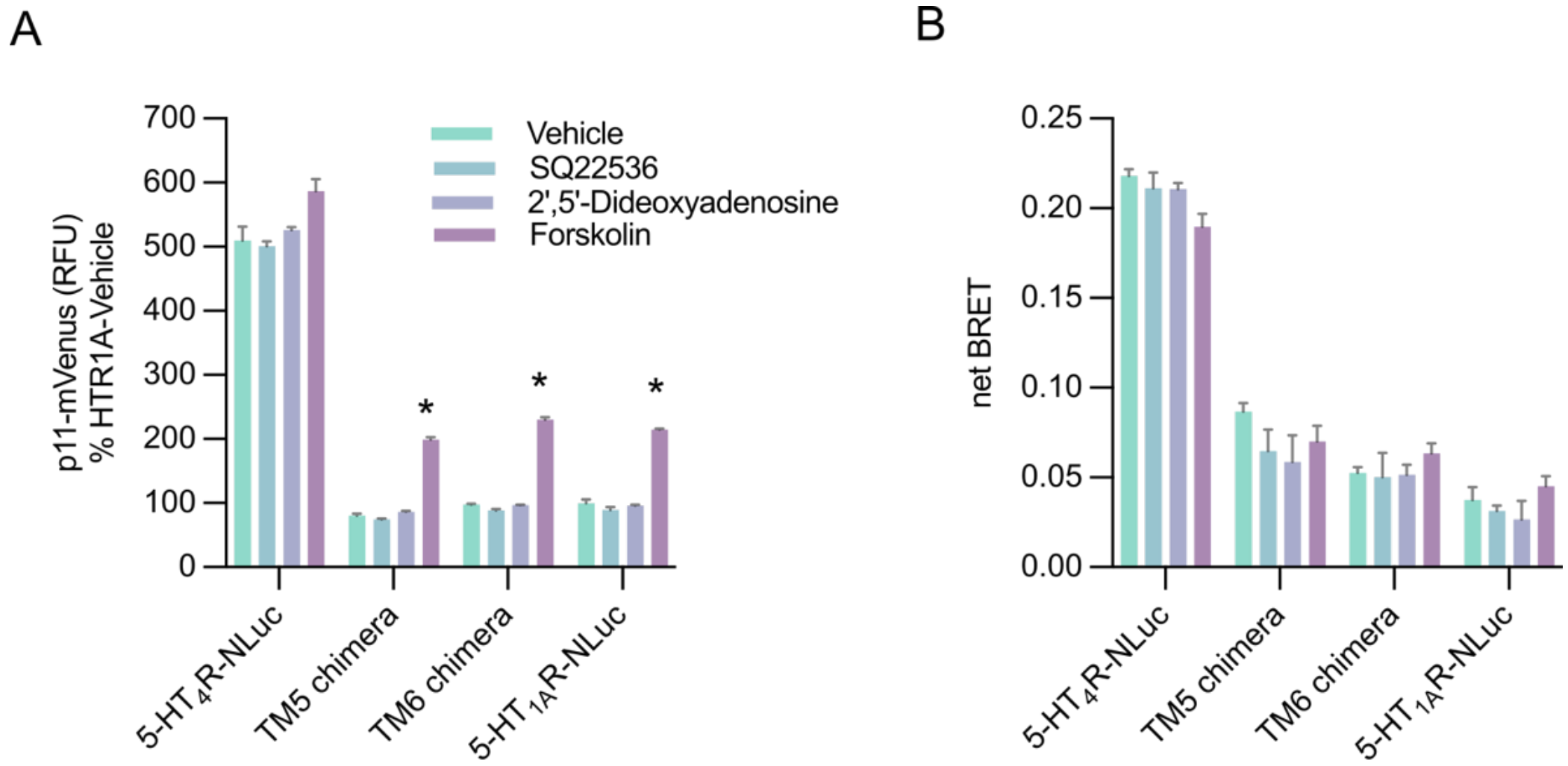

Fig. S6: Effects of cAMP on p11-mVenus abundance and BRET in p11-KO cells
(A) p11-mVenus fluorescence counts and **(B)** netBRET from p11-mVenus (1 pg DNA/cell) and receptor-NLuc (0.002 pg DNA/cell) co-transfected in p11-KO cells and treated with AC stimulator forskolin (1µM), AC inhibitor SQ22536 (100µM) or AC inhibitor 2',5'-Dideoxyadenosine (50µM) for 4 (*n*=3 technical replicates, mean + SEM) (*$p<0.05$, Forskolin vs vehicle, 2-Way ANOVA with Tukey's multiple comparison test)

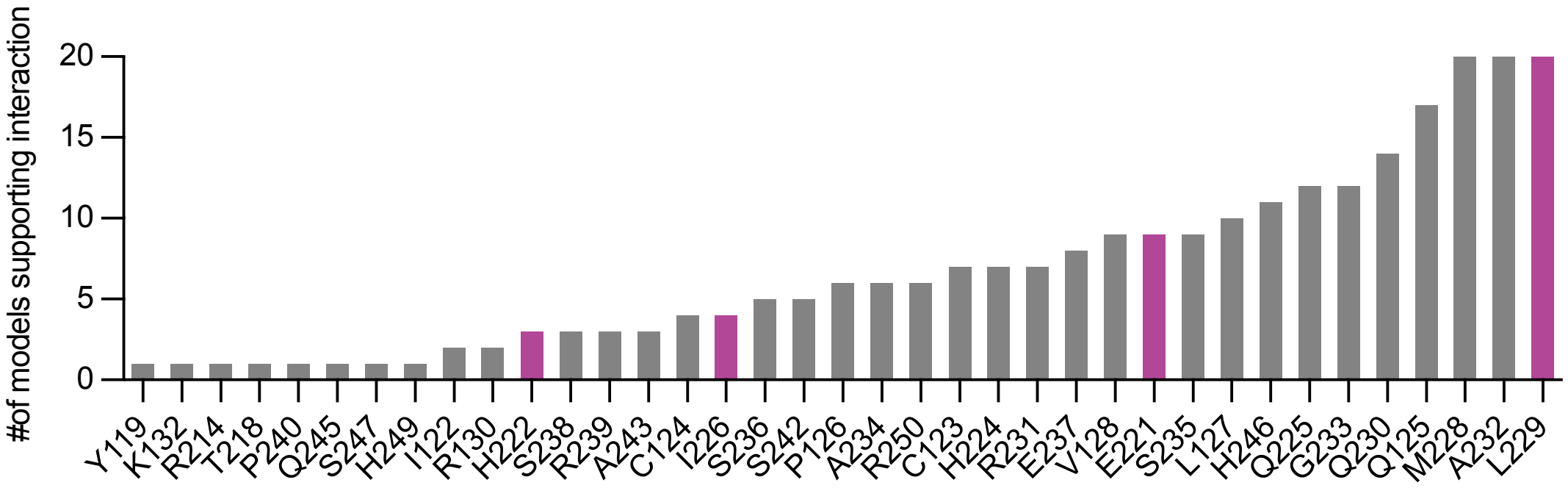

Fig. S7: AF3 predicted 5-HT_4R residues forming an interface with p11

Residues are ranked by the number of models which predict the residue is involved in an interface with p11. Purple colored bars represent 5-HT_4R-NLuc residues identified to modulate p11-mVenus levels or net-BRET in mutagenesis experiments outlined in Figure 2C.

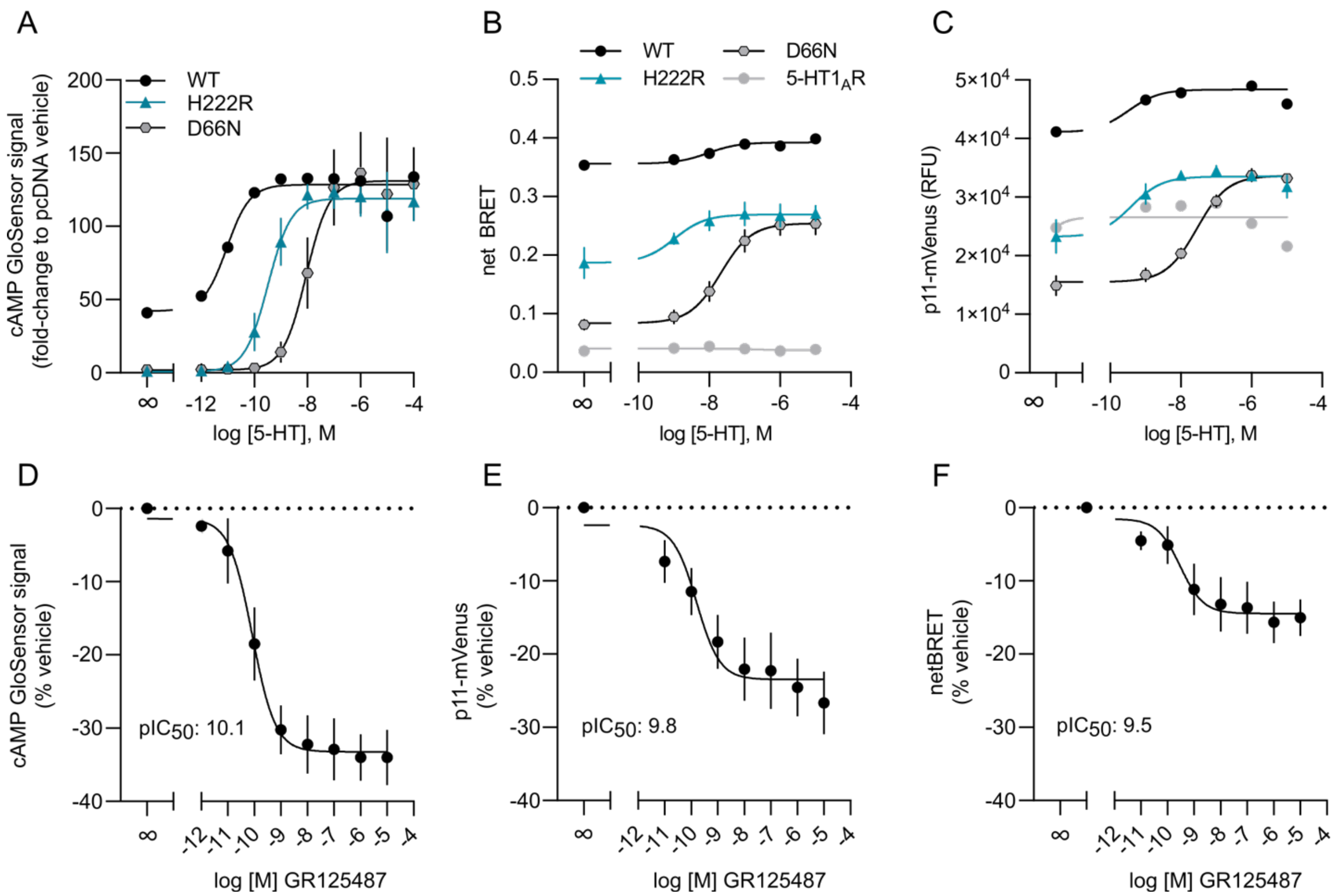

Fig. S8: Assessment of receptor signaling profiles
(A) Concentration-response curves of the signal from cAMP sensor (0.2pg DNA/cell) co-expressed with 5-HT_4R WT-NLuc, 5-HT_4R H222R-NLuc or 5-HT_4R D66N-NLuc (0.083 pg receptor DNA/cell) treated with 5-HT. (*n*=3, mean ± SEM).
(B) netBRET from p11-mVenus (1pg DNA/cell) co-expressed with 5-HT_4R WT-NLuc, 5-HT_4R H222R-NLuc or 5-HT_4R D66N-NLuc (0.002pg DNA/cell) after overnight treatment with ligand 5-HT. (*n*=3, mean ± SEM).
(C) p11-mVenus signal cells transfected with p11-mVenus (1pg DNA/cell) and 5-HT_4R WT-NLuc, 5-HT_4R H222R-NLuc or 5-HT_4R D66N-NLuc (0.002pg DNA/cell) after overnight treatment with ligand 5-HT. (*n*=3, mean ± SEM).
(D) Partial inhibition of baseline cAMP in 5-HT_4R-NLuc expressing Expi293F cells using the inverse agonist GR125487. Error bars indicate mean ± SEM.
(E-F) Concentration-dependent, partial decrease of p11-mVenus fluorescence (E) and netBRET (F) between 5-HT_4R-NLuc p11-mVenus. Each point represents three independent experiments, error bars indicate mean ± SEM. pIC50 values were obtained using a three-parameter, nonlinear regression curve analysis.

Values in Figures 2E-G correspond to the baseline values in Fig. S5A-C, respectively.

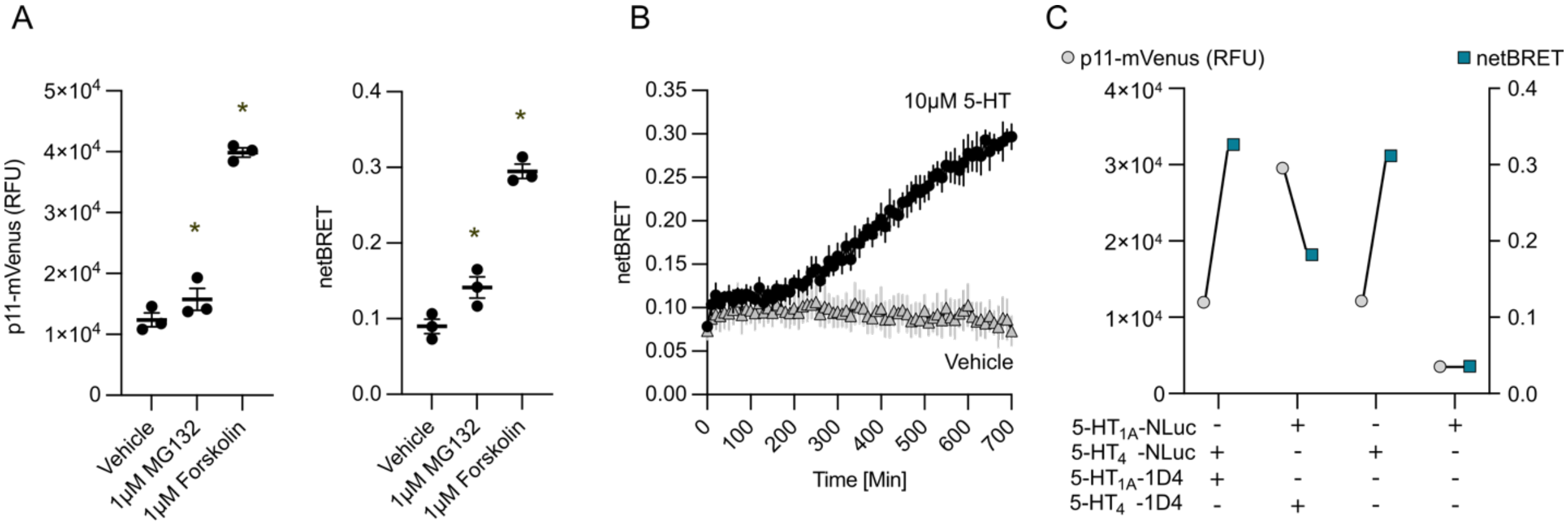

Fig. S9: Further characterization of p11 recruitment BRET assay

(A) *Left:* p11-mVenus fluorescence counts and *Right:* netBRET measurements from p11-mVenus (1 pg DNA/cell) and 5-HT$_4$R-D66N-NLuc (0.002 pg DNA/cell) co-transfected in p11-KO cells (*n*=3, mean ± SEM) (*p<0.05, comparison to vehicle, One-way ANOVA, Dunnett's multiple comparisons test)

(B) netBRET measurements from p11-mVenus (1 pg DNA/cell) and 5-HT$_4$R-D66N-NLuc (0.002 pg DNA/cell) co-transfected in p11-KO cells treated with 5-HT or vehicle. Treatment was done at time point 0. (*n*=3, mean ± SEM)

(C) Trans-activation assays of either DuET or NLuc 5-HT$_4$R or 5-HT$_{1A}$R (0.002 pg DNA/cell) co-expressed with p11-mVenus (1 pg DNA/cell). Each point represents the mean of three individual experiments.

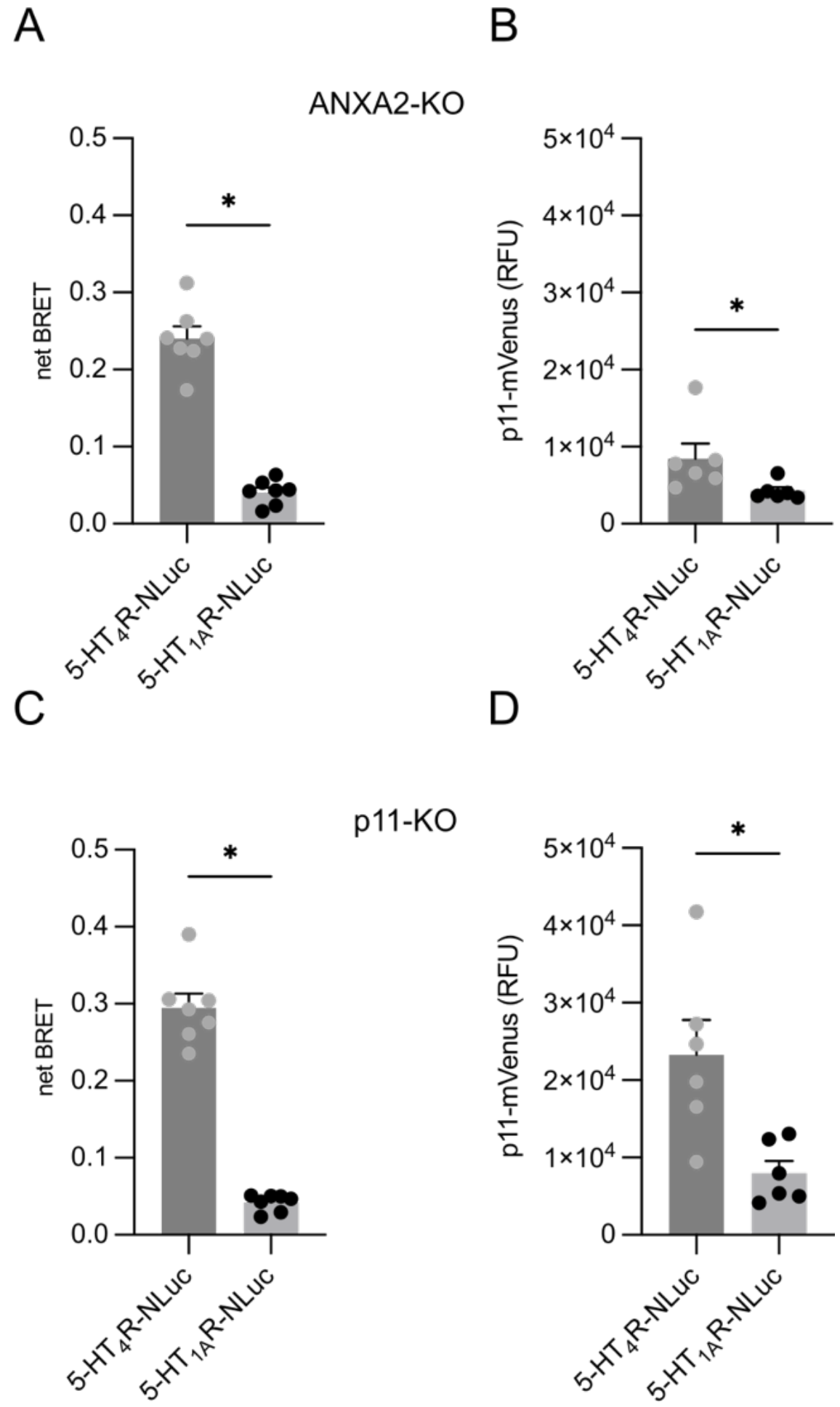

Fig. S10: p11-mVenus abundance in p11-KO and ANXA2-KO cells

(A) netBRET measurements, and **(B)** p11-mVenus fluorescence counts from p11-mVenus (1 pg DNA/cell) and 5-HT_4R-NLuc or 5-HT_{1A}R-NLuc (0.002 pg DNA/cell) co-transfected in ANXA2-KO cells (*n*=6-7, mean + SEM) (*$p<0.05$, paired t-test and Wilcoxon test, respectively).

(C) netBRET measurements, and **(D)** p11-mVenus fluorescence counts from p11-mVenus (1 pg DNA/cell) and 5-HT_4R-NLuc or 5-HT_{1A}R-NLuc (0.002 pg DNA/cell) co-transfected in p11-KO cells (*n*=6-7, mean + SEM) (*$p<0.05$, paired t-test)

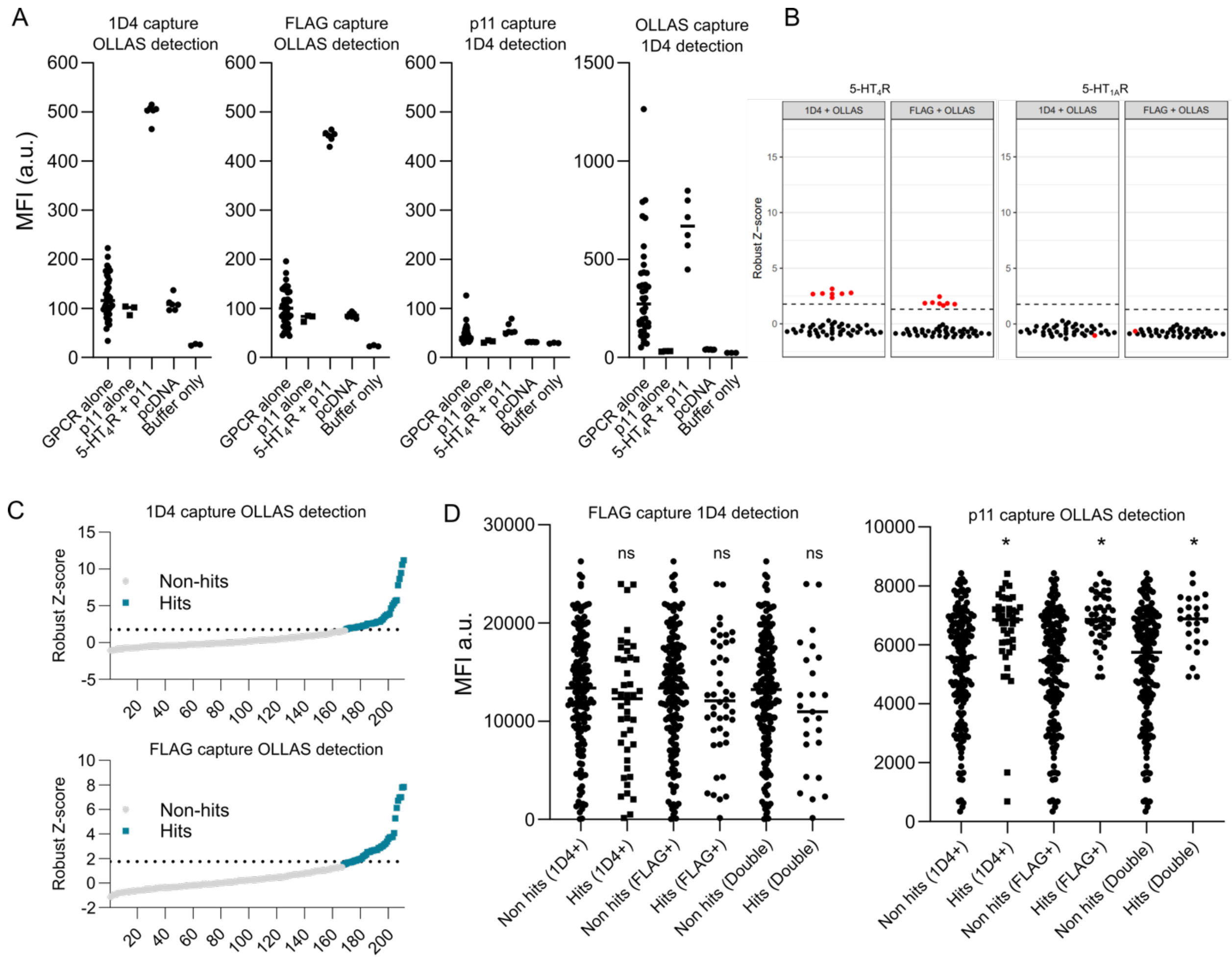

Fig. S11: Evaluation of the SBA to interrogate GPCR-p11 complexes

(A) Dot plots of all four capture-detection schemes. Y axis represents mean fluorescence intensity with each dot representing one sample. Lines represent median, (a.u.: arbitrary units)

(B) Beeswarm plot showing the robust Z-score of the MFI for each capture detection scheme for 40 GPCR-no p11 transfected samples (black dots) and the highlighted receptor (red dots). The dashed line indicates the statistical cutoff used to evaluate hits

(C) Representation of the hit curve for the FLAG capture, OLLAS detection scheme and the 1D4 capture, OLLAS detection scheme, respectively. Each dot on the plot signifies a single receptor-p11 co-expressing sample, ranked by the robust Z-score of the MFI for that sample on the y-axis. Dotted line indicates statistical cutoff.

(D) Dot plots displaying the MFI values from each capture-detection scheme for hits and non-hits. Left graph depicts FLAG capture, 1D4 detection MFI values, which are used to infer GPCR expression levels, while in the right panel, OLLAS capture p11 detection MFI values are utilized for quantitation of p11 abundance in each group. (ns= not significant, *$p<0.05$*, Brown-Forsythe and Welch ANOVA with Dunnet's T3 multiple comparison test), lines represent median.

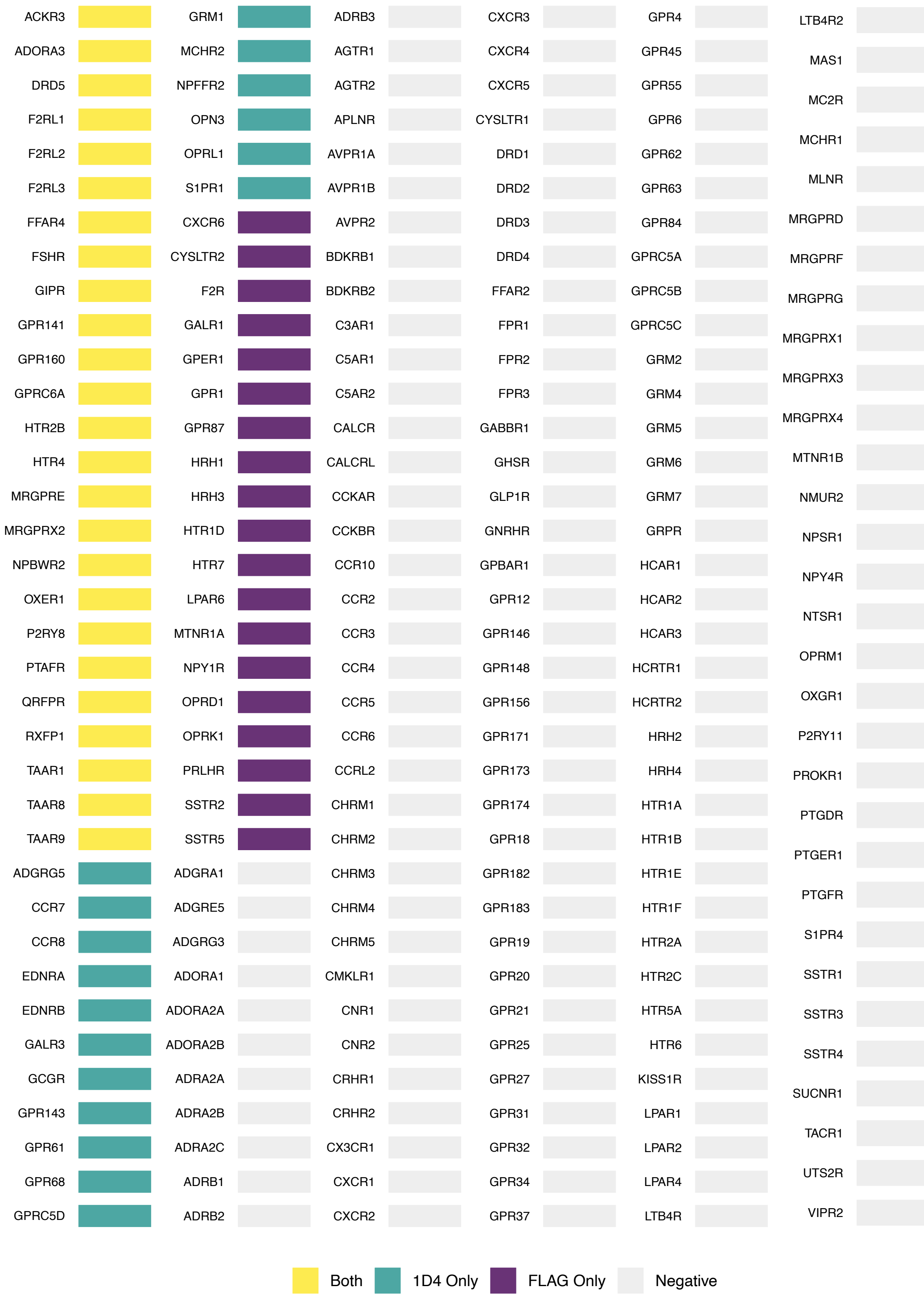

Fig. S12: All tested GPCR-p11 pairs in the SBA

Heat map of all DuET GPCRs co-expressed with p11-OLLAS, colored by the number of capture-detection schemes they passed.

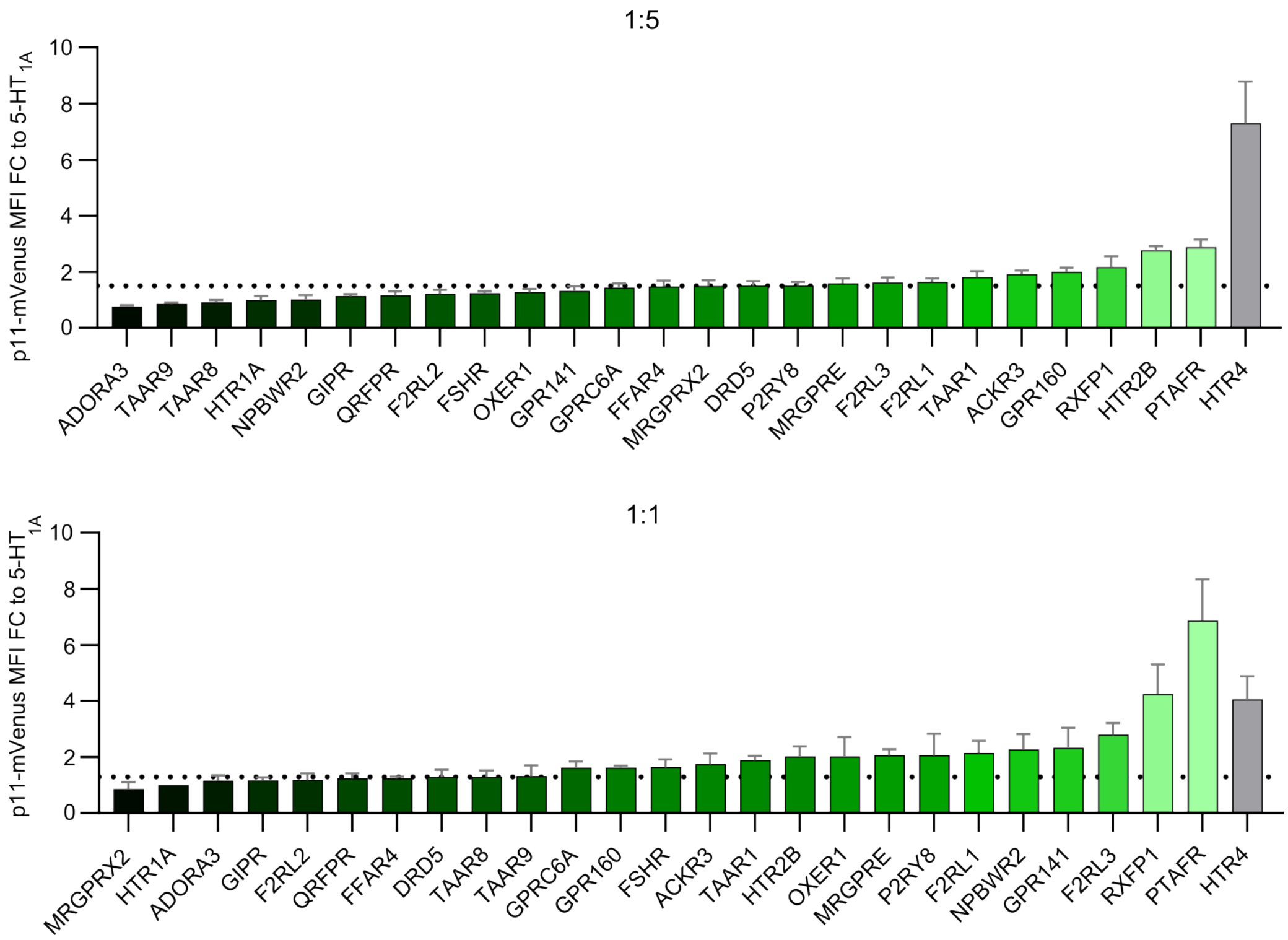

Fig. S13: p11-mVenus levels upon co-transfection with double positive hits
p11-mVenus was measured in each well of a transparent bottom, black 96-well plate after well confluency determination. Bar plots display the adjusted MFI from each p11-GPCR co-transfected pair normalized to the MFI of 5-HT_{1A}R-p11 in the same plate. Results shown in the top bar plot are from a 1 to 5 gene dose of GPCR:p11 while the bottom shows the results from 1 to 1 transfected samples. Dotted line indicates mean+3*S.D of the 5-HT_{1A}R used as a cutoff. Each bar shows the mean + SEM of 3-5 biological replicates.

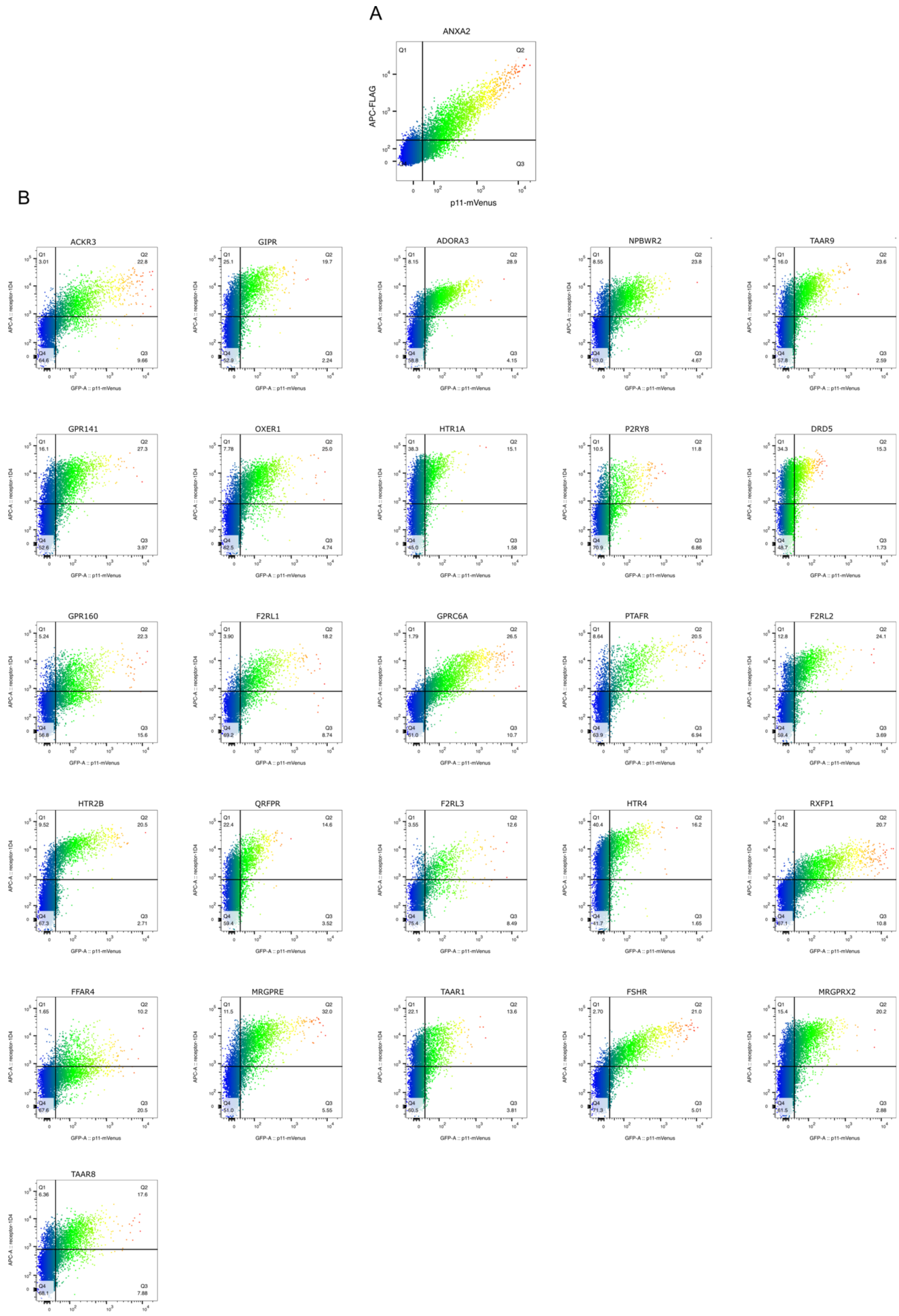

Fig. S14: Flow cytometry enabled quantitation of p11-mVenus with ANXA2 or double-positive hits from the SBA

(A) ANXA2-FLAG transfected p11-KO cells were stained using APC-FLAG and subjected to flow cytometry. APC signal (y axis) indicates ANXA2 abundance and mVenus fluorescence (p11) is shown on the x-axis. Units indicate MFI.
(B) Plots of double-positive SBA hits co-expressed with p11-mVenus in p11-KO cells. Each plot is taken from one biological replicate. Left upper quarter: 1D4 (GPCR) positive, p11-mVenus negative population. Upper right quarter: 1D4 and p11-mVenus positive population. Lower right quarter: p11-mVenus positive, 1D4 negative population. Lower left quarter: 1D4 and p11-mVenus negative population

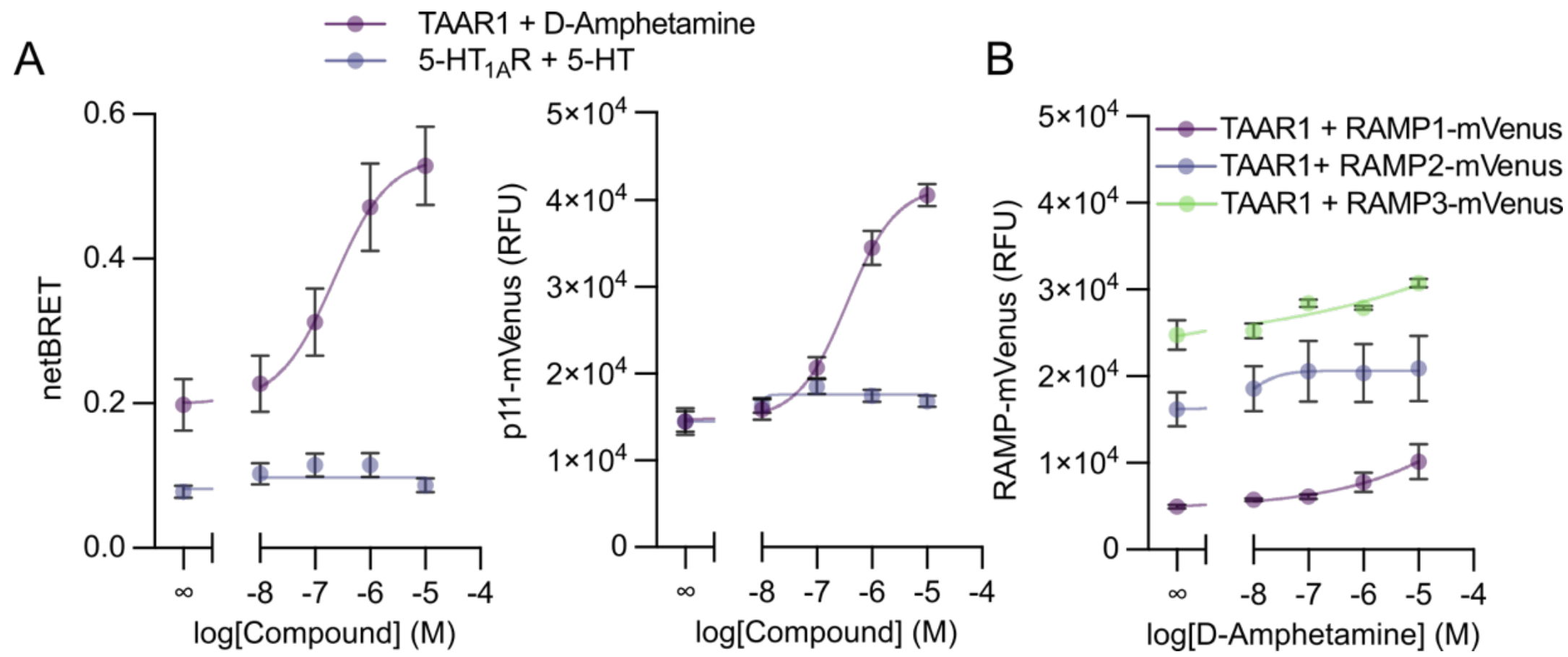

Fig. S15: Control mVenus construct induction by TAAR1 activation

(A) TAAR1-NLuc (0.02pg DNA/cell)or 5-HT1AR-NLuc (0.002pg DNA/cell) were transfected in p11-KO cells together with p11-mVenus (1pg DNA/cell). BRET (left) and p11-mVenus (right) measurements were performed following overnight treatment with D-amphetamine or 5-HT.

(B) mVenus measurements from HEK293T cells co-transfected with TAAR1-NLuc (0.02pg DNA/cell) and RAMP1, 2 or 3-mVenus (1pg DNA/cell) constructs, treated overnight with D-amphetamine.

Error bars show mean ± SEM of three biological replicates.

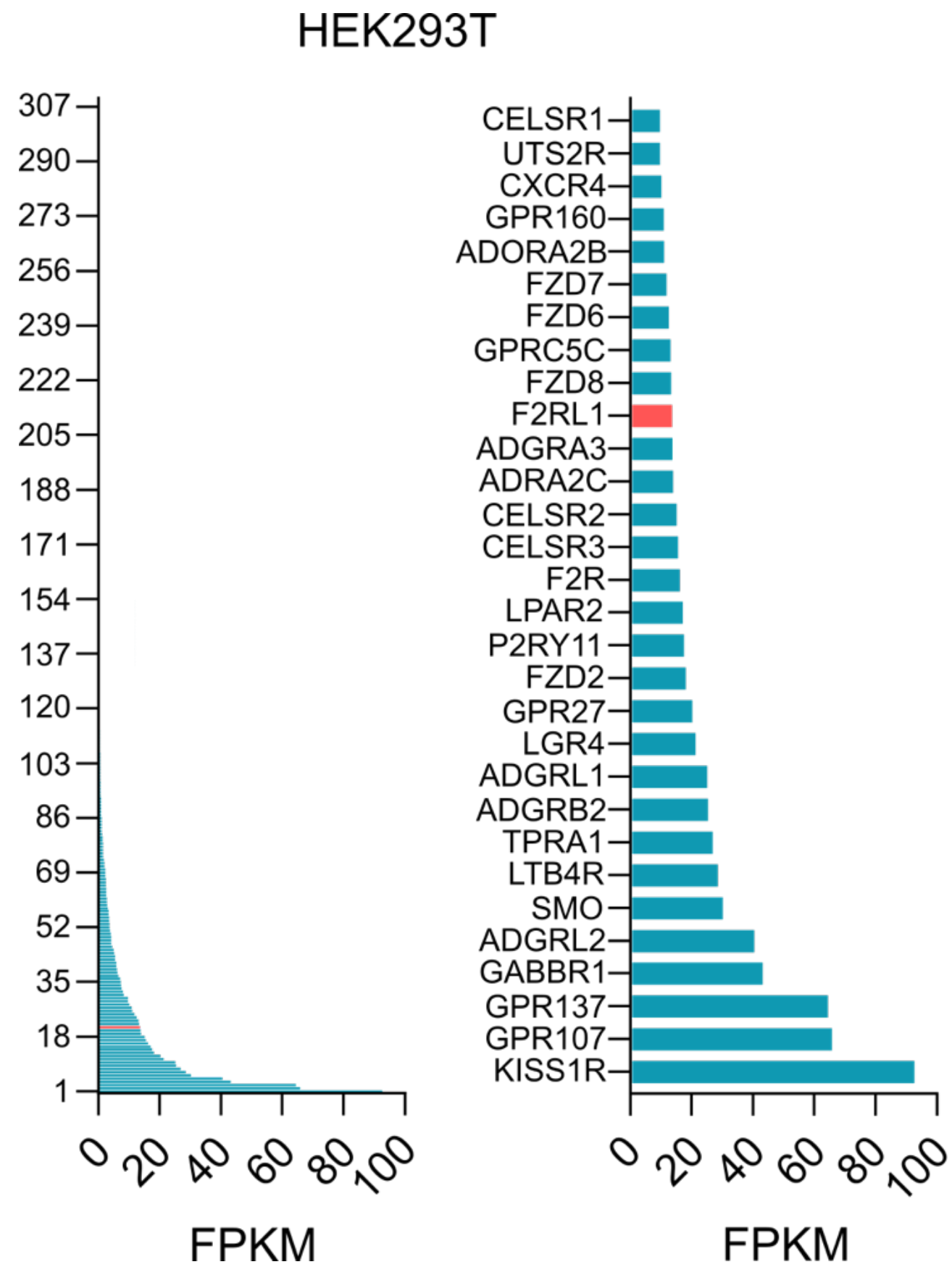

Fig. S16: GPCR gene transcript counts from HEK293T cells
GPCR gene IDs were mapped to RNA sequencing data from (52), ranked by expression. Left panel shows FPKM counts for all GPCR IDs, with the top 30 shown on the right panel. PAR2 (*F2RL1)*, is highlighted in red.

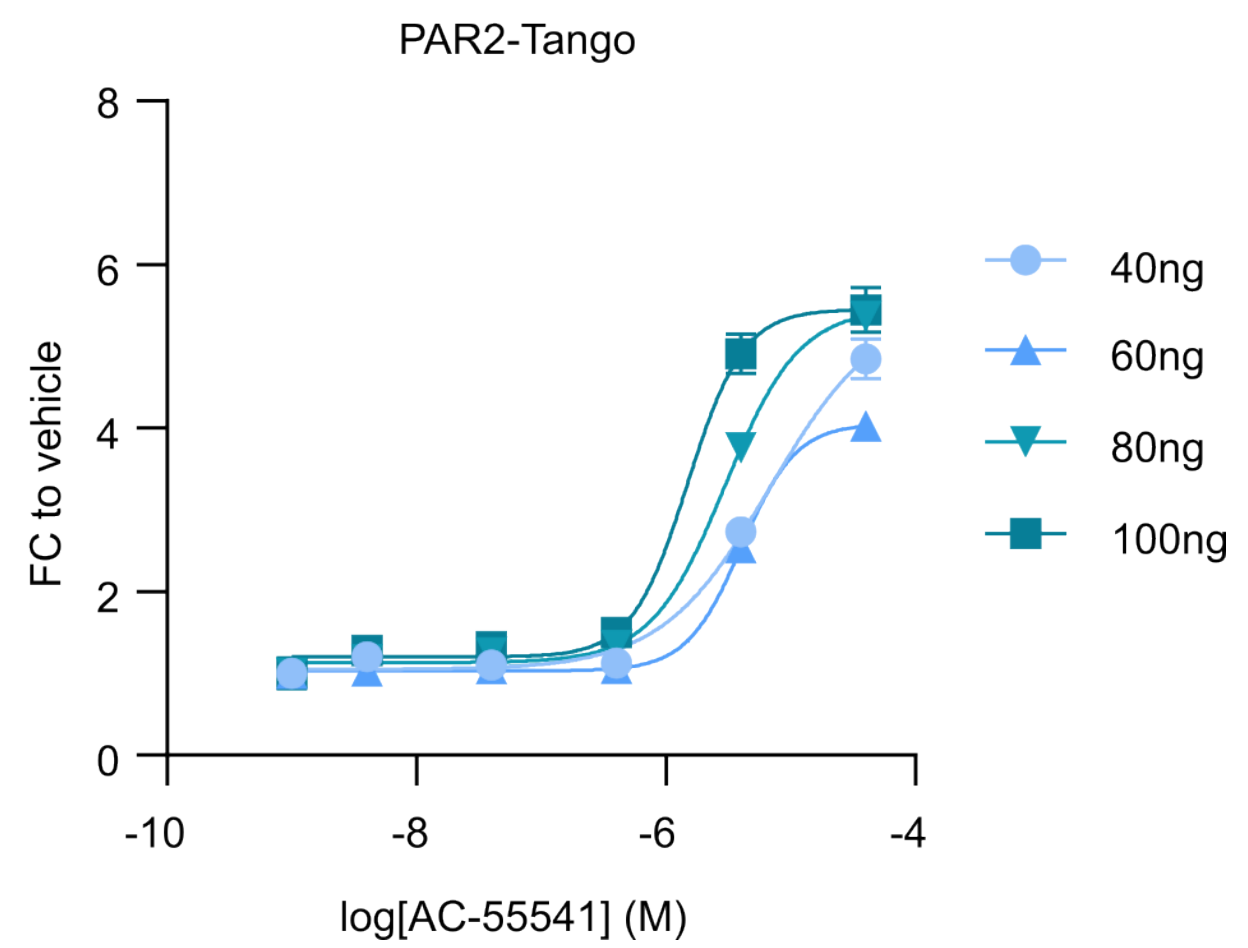

Fig. S17: AC-55541 response to various PAR2 gene doses
HTLA cells were transfected in 96-well plates with the indicated amount of PAR2-Tango construct with total gene dose balanced to 100ng/well using empty plasmid. Luminescence from PAR2 activation by AC-55541 was measured following overnight stimulation. Each point shows the mean ± SEM of 4 technical replicates.

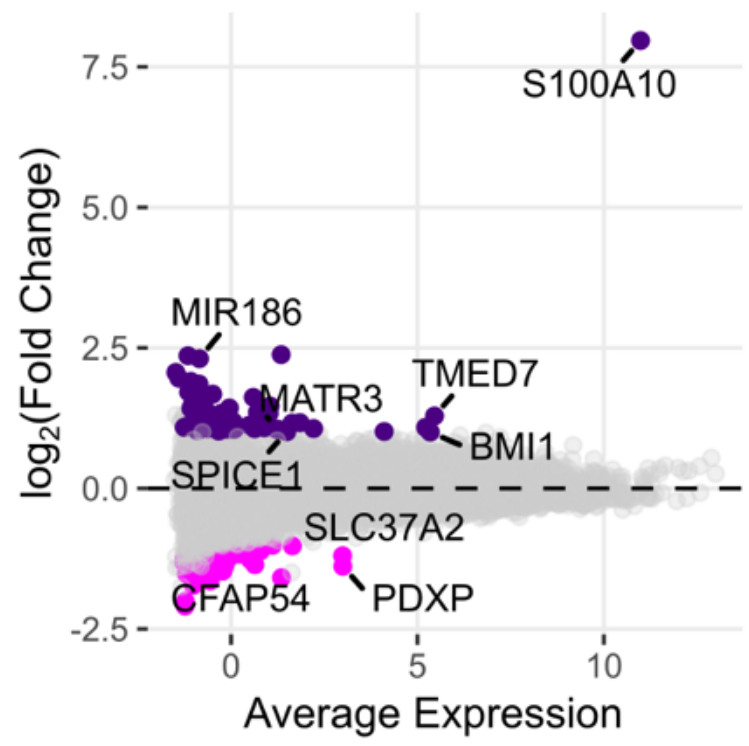

Fig. S18: MA-plot of baseline affected genes
Significantly up- or down-regulated genes in HEK293T p11-KO cells transfected with either empty plasmid (pcDNA) or p11-mVenus. Only genes with a log(FC)>1 or <-1 are colored for clarity.

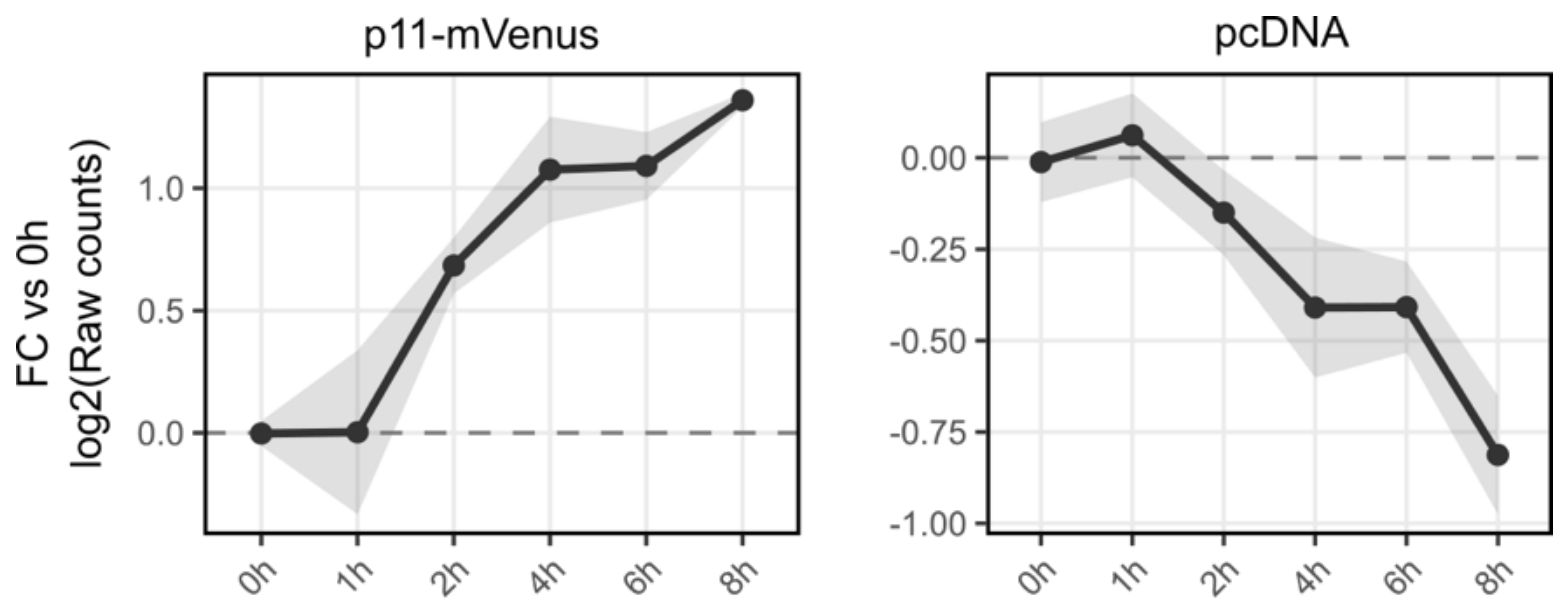

Fig. S19: *S100A10* expression following AC-55541 stimulation
Raw counts (shown in a log2 scale for clarity, FC fold-change) of *S100A10* for p11-Venus samples (left) and pcDNA samples (right) at each time-point.

Error bars show mean ± SEM

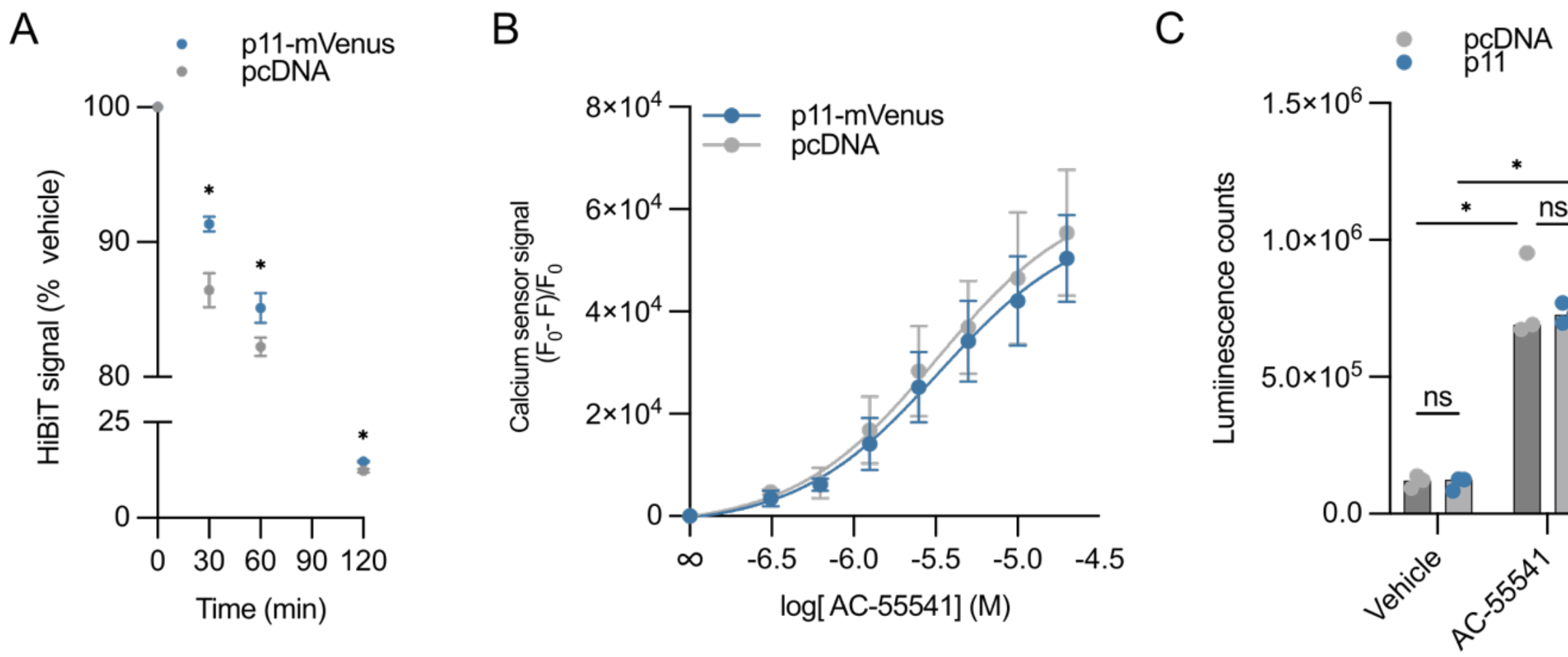

Fig.S20: PAR2 surface expression and signaling in the presence or absence of p11
(**A)** Surface expression of N-terminally HiBiT tagged PAR2-mScarlet3 (0.002 pg DNA/cell) assessed using the Nano-Glo HiBiT Extracellular Detection System, with or without p11-mVenus (1 pg DNA/cell) co-transfected in p11-KO cells, with or without AC-55541 (10µM). (*n*=3, mean ± SEM) (*p<0.05, p11-mVenus vs pcDNA, RM Two-way ANOVA, Tukey's multiple comparison test).
(**B)** Calcium sensor signal upon endogenous PAR2 activation by AC-55541 in p11-KO cells, with or without p11-mVenus transfection (1pg DNA/cell). (*n*=3 mean ± SEM).
(C) ß-Arrestin recruitment to PAR2-Tango (1.6pg DNA/cell) in response to vehicle or AC-55541 (4µM) assessed using the PRESTO-TANGO method, with or without p11-OLLAS (0.4pg DNA/cell) co-transfected in HTLA cells (*n*=3) (*p<0.05, RM Two-way ANOVA, Uncorrected Fisher's LSD).

Table S1: DEGs in p11-mVenus or pcDNA transfected p11-KO cells

The workbook contains the full lists of DEGs for the baseline comparison between p11-mVenus and pcDNA control cells at 0h, as well as the temporal response of both cell lines to AC-55541 across an 8-hour time course relative to their respective 0h baselines.

REFERENCES

1. M. C. Lagerström, H. B. Schiöth, Structural diversity of G protein-coupled receptors and significance for drug discovery. *Nat. Rev. Drug Discov.* **7**, 339–357 (2008).

2. K. Sriram, P. A. Insel, G protein-coupled receptors as targets for approved drugs: How many targets and how many drugs?. *Mol. Pharmacol.* **93**, 251–258 (2018).

3. B. K. Kobilka, G protein coupled receptor structure and activation. *Biochim. Biophys. Acta* **1768**, 794–807 (2007).

4. S. L. Ritter, R. A. Hall, Fine-tuning of GPCR activity by receptor-interacting proteins. *Nat. Rev. Mol. Cell Biol.* **10**, 819–830 (2009).

5. L. Yuan, S. Barbash, S. Kongsamut, A. Eishingdrelo, T. P. Sakmar, H. Eishingdrelo, 14-3-3 signal adaptor and scaffold proteins mediate GPCR trafficking. *Sci. Rep.* **9**, 11156 (2019).

6. I. B. Kotliar, E. Lorenzen, J. M. Schwenk, D. L. Hay, T. P. Sakmar, Elucidating the interactome of G protein-coupled receptors and receptor activity-modifying proteins. *Pharmacol. Rev.* **75**, 1–34 (2023).

7. E. L. Moore, C. A. Salvatore, Targeting a family B GPCR/RAMP receptor complex: CGRP receptor antagonists and migraine. *Br. J. Pharmacol.* **166**, 66–78 (2012).

8. V. Gerke, K. Weber, Identity of p36K phosphorylated upon Rous sarcoma virus transformation with a protein purified from brush borders; calcium-dependent binding to non-erythroid spectrin and F-actin. *EMBO J.* **3**, 227–233 (1984).

9. P. Svenningsson, K. Chergui, I. Rachleff, M. Flajolet, X. Zhang, M. E. Yacoubi, J.-M. Vaugeois, G. G. Nomikos, P. Greengard, Alterations in 5-HT_{1B} receptor function by p11 in depression-like states. *Science* **311**, 77–80 (2006).

10. J. L. Warner-Schmidt, M. Flajolet, A. Maller, E. Y. Chen, H. Qi, P. Svenningsson, P. Greengard, Role of p11 in cellular and behavioral effects of 5-HT4 receptor stimulation. *J. Neurosci.* **29**, 1937–1946 (2009).

11. B. D. Cohen, C. A. Nechamen, J. A. Dias, Human follitropin receptor (FSHR) interacts with the adapter protein 14-3-3τ. *Mol. Cell. Endocrinol.* **220**, 1–7 (2004).

12. K.-W. Lee, L. Westin, J. Kim, J. C. Chang, Y.-S. Oh, B. Amreen, J. Gresack, M. Flajolet, D. Kim, A. Aperia, Y. Kim, P. Greengard, Alteration by p11 of mGluR5 localization regulates depression-like behaviors. *Mol. Psychiatry* **20**, 1546–1556 (2015).

13. F. Hessner, C. P. Dlugos, T. Chehab, C. Schaefer, B. Homey, V. Gerke, T. Weide, H. Pavenstädt, U. Rescher, CC chemokine receptor 10 cell surface presentation in melanocytes is regulated by the novel interaction partner S100A10. *Sci. Rep.* **6**, 22649 (2016).

14. M. Karlsson, C. Zhang, L. Méar, W. Zhong, A. Digre, B. Katona, E. Sjöstedt, L. Butler, J. Odeberg, P. Dusart, F. Edfors, P. Oksvold, K. Von Feilitzen, M. Zwahlen, M. Arif, O. Altay, X. Li, M. Ozcan, A. Mardinoglu, L. Fagerberg, J. Mulder, Y. Luo, F. Ponten, M. Uhlén, C. Lindskog, A single–cell type transcriptomics map of human tissues. *Sci. Adv.* **7**, eabh2169 (2021).

15. M. Uhlen, C. Zhang, S. Lee, E. Sjöstedt, L. Fagerberg, G. Bidkhori, R. Benfeitas, M. Arif, Z. Liu, F. Edfors, K. Sanli, K. Von Feilitzen, P. Oksvold, E. Lundberg, S. Hober, P. Nilsson, J. Mattsson, J. M. Schwenk, H. Brunnström, B. Glimelius, T. Sjöblom, P.-H. Edqvist, D. Djureinovic, P. Micke, C. Lindskog, A. Mardinoglu, F. Ponten, A pathology atlas of the human cancer transcriptome. *Science* **357**, eaan2507 (2017).

16. H. Lu, Y. Xie, L. Tran, J. Lan, Y. Yang, N. L. Murugan, R. Wang, Y. J. Wang, G. L. Semenza, Chemotherapy-induced S100A10 recruits KDM6A to facilitate OCT4-mediated breast cancer stemness. *J. Clin. Invest.* **130**, 4607–4623 (2020).

17. Y. Laumonnier, T. Syrovets, L. Burysek, T. Simmet, Identification of the annexin A2 heterotetramer as a receptor for the plasmin-induced signaling in human peripheral monocytes. *Blood* **107**, 3342–3349 (2006).

18. G. Kassam, B.-H. Le, K.-S. Choi, H.-M. Kang, S. L. Fitzpatrick, P. Louie, D. M. Waisman, The p11 subunit of the annexin II tetramer plays a key role in the stimulation of t-PA-dependent plasminogen activation. *Biochemistry* **37**, 16958–16966 (1998).

19. Y. Xiao, H. Zhao, L. Tian, R. Nolley, A. N. Diep, A. Ernst, K. C. Fuh, Y. R. Miao, R. Von Eyben, J. T. Leppert, J. D. Brooks, D. M. Peehl, A. J. Giaccia, E. B. Rankin, S100A10 is a critical mediator of GAS6/AXL–induced angiogenesis in renal cell carcinoma. *Cancer Res.* **79**, 5758–5768 (2019).

20. I. B. Kotliar, A. Bendes, L. Dahl, Y. Chen, M. Saarinen, E. Ceraudo, T. Dodig-Crnković, M. Uhlén, P. Svenningsson, J. M. Schwenk, T. P. Sakmar, Multiplexed mapping of the interactome of GPCRs with receptor activity–modifying proteins. *Sci. Adv.* **10**, eado9959 (2024).

21. K.-L. He, A. B. Deora, H. Xiong, Q. Ling, B. B. Weksler, R. Niesvizky, K. A. Hajjar, Endothelial cell annexin A2 regulates polyubiquitination and degradation of its binding partner S100A10/p11. *J. Biol. Chem.* **283**, 19192–19200 (2008).

22. A. B. Deora, G. Kreitzer, A. T. Jacovina, K. A. Hajjar, An annexin 2 phosphorylation switch mediates p11-dependent translocation of annexin 2 to the cell surface. *J. Biol. Chem.* **279**, 43411–43418 (2004).

23. N. H. Cho, K. C. Cheveralls, A.-D. Brunner, K. Kim, A. C. Michaelis, P. Raghavan, H. Kobayashi, L. Savy, J. Y. Li, H. Canaj, J. Y. S. Kim, E. M. Stewart, C. Gnann, F. McCarthy, J. P. Cabrera, R. M. Brunetti, B. B. Chhun, G. Dingle, M. Y. Hein, B. Huang, S. B. Mehta, J. S. Weissman, R. Gómez-Sjöberg, D. N. Itzhak, L. A. Royer, M. Mann, M. D. Leonetti, OpenCell: Endogenous tagging for the cartography of human cellular organization. *Science* **375**, eabi6983 (2022).

24. S. Huang, P. Xu, D.-D. Shen, I. A. Simon, C. Mao, Y. Tan, H. Zhang, K. Harpsøe, H. Li, Y. Zhang, C. You, X. Yu, Y. Jiang, Y. Zhang, D. E. Gloriam, H. E. Xu, GPCRs steer G_i and G_s selectivity via TM5-TM6 switches as revealed by structures of serotonin receptors. *Mol. Cell* **82**, 2681–2695.e6 (2022).

25. K. L. White, M. T. Eddy, Z.-G. Gao, G. W. Han, T. Lian, A. Deary, N. Patel, K. A. Jacobson, V. Katritch, R. C. Stevens, Structural connection between activation microswitch and allosteric sodium site in GPCR signaling. *Structure* **26**, 259–269.e5 (2018).

26. S. Claeysen, M. Sebben, C. Bécamel, R. M. Eglen, R. D. Clark, J. Bockaert, A. Dumuis, Pharmacological properties of 5-hydroxytryptamine$_4$ receptor antagonists on constitutively active wild-type and mutated receptors. *Mol. Pharmacol.* **58**, 136–144 (2000).

27. C. J. Peach, L. E. Edgington-Mitchell, N. W. Bunnett, B. L. Schmidt, Protease-activated receptors in health and disease. *Physiol. Rev.* **103**, 717–785 (2023).

28. N. Vergnolle, N. W. Bunnett, K. A. Sharkey, V. Brussee, S. J. Compton, E. F. Grady, G. Cirino, N. Gerard, A. I. Basbaum, P. Andrade-Gordon, M. D. Hollenberg, J. L. Wallace, Proteinase-activated receptor-2 and hyperalgesia: A novel pain pathway. *Nat. Med.* **7**, 821–826 (2001).

29. L. R. Gardell, J.-N. Ma, J. G. Seitzberg, A. E. Knapp, H. H. Schiffer, A. Tabatabaei, C. N. Davis, M. Owens, B. Clemons, K. K. Wong, B. Lund, N. R. Nash, Y. Gao, J. Lameh, K. Schmelzer, R. Olsson, E. S. Burstein, Identification and characterization of novel small-molecule protease-activated receptor 2 agonists. *J. Pharmacol. Exp. Ther.* **327**, 799–808 (2008).

30. G. Pándy-Szekeres, L. P. T. Herrera, J. Caroli, A. A. Kermani, Y. Kulkarni, G. M. Keserű, D. E. Gloriam, GproteinDb in 2024: New G protein-GPCR couplings, AlphaFold2-multimer models and interface interactions. *Nucleic Acids Res.* **52**, D466–D475 (2024).

31. M. Steinhoff, N. Vergnolle, S. H. Young, M. Tognetto, S. Amadesi, H. S. Ennes, M. Trevisani, M. D. Hollenberg, J. L. Wallace, G. H. Caughey, S. E. Mitchell, L. M. Williams, P. Geppetti, E. A. Mayer, N. W. Bunnett, Agonists of proteinase-activated receptor 2 induce inflammation by a neurogenic mechanism. *Nat. Med.* **6**, 151–158 (2000).

32. S. N. Hassler, M. Kume, J. M. Mwirigi, A. Ahmad, S. Shiers, A. Wangzhou, P. R. Ray, S. N. Belugin, D. K. Naik, M. D. Burton, J. Vagner, S. Boitano, A. N. Akopian, G. Dussor, T. J. Price, The cellular basis of protease-activated receptor 2–evoked mechanical and affective pain. *JCI Insight* **5**, e137393 (2020).

33. C.-L. Li, K.-C. Li, D. Wu, Y. Chen, H. Luo, J.-R. Zhao, S.-S. Wang, M.-M. Sun, Y.-J. Lu, Y.-Q. Zhong, X.-Y. Hu, R. Hou, B.-B. Zhou, L. Bao, H.-S. Xiao, X. Zhang, Somatosensory neuron types identified by high-coverage single-cell RNA-sequencing and functional heterogeneity. *Cell Res.* **26**, 83–102 (2016).

34. I. Brandherm, J. Disse, D. Zeuschner, V. Gerke, cAMP-induced secretion of endothelial von Willebrand factor is regulated by a phosphorylation/dephosphorylation switch in annexin A2. *Blood* **122**, 1042–1051 (2013).

35. T. Chehab, N. C. Santos, A. Holthenrich, S. N. Koerdt, J. Disse, C. Schuberth, A. R. Nazmi, M. Neeft, H. Koch, K. N. M. Man, S. M. Wojcik, T. F. J. Martin, P. Van Der Sluijs, N. Brose, V. Gerke, A novel Munc13-4/S100A10/annexin A2 complex promotes Weibel–Palade body exocytosis in endothelial cells. *Mol. Biol. Cell* **28**, 1688–1700 (2017).

36. E. A. Peterson, M. R. Sutherland, M. E. Nesheim, E. L. G. Pryzdial, Thrombin induces endothelial cell-surface exposure of the plasminogen receptor annexin 2. *J. Cell Sci.* **116**, 2399–2408 (2003).

37. X. Zhong, Q. Li, B. J. Polacco, T. Patil, A. Marley, H. Foussard, P. Khare, R. Vartak, J. Xu, J. F. DiBerto, B. L. Roth, M. Eckhardt, M. Von Zastrow, N. J. Krogan, R. Hüttenhain, A proximity proteomics pipeline with improved reproducibility and throughput. *Mol. Syst. Biol.* **20**, 952–971 (2024).

38. M. M. Shchepinova, R. Richardson, J. W. Houghton, A. R. Walker, M. A. Safar, D. Conole, A. C. Hanyaloglu, E. W. Tate, Spatiotemporally resolved GPCR interactome uncovers unique mediators of receptor agonism. *Cell Chem. Biol.* **32**, 722–735.e7 (2025).

39. N. Stroth, P. Svenningsson, S100B interacts with the serotonin 5-HT7 receptor to regulate a depressive-like behavior. *Eur. Neuropsychopharmacol.* **25**, 2372–2380 (2015).

40. Y. Liu, D. C. Buck, K. A. Neve, Novel interaction of the dopamine D_2 receptor and the Ca^{2+} binding protein S100B: Role in D_2 receptor function. *Mol. Pharmacol.* **74**, 371–378 (2008).

41. A. B. Kleist, S. Jenjak, A. Sente, L. J. Laskowski, M. Szpakowska, M. M. Calkins, E. I. Anderson, L. M. McNally, R. Heukers, V. Bobkov, F. C. Peterson, M. A. Thomas, A. Chevigné, M. J. Smit, J. D. McCorvy, M. M. Babu, B. F. Volkman, Conformational selection guides β-arrestin recruitment at a biased G protein–coupled receptor. *Science* **377**, 222–228 (2022).

42. S. Rajagopal, J. Kim, S. Ahn, S. Craig, C. M. Lam, N. P. Gerard, C. Gerard, R. J. Lefkowitz, β-Arrestin- but not G protein-mediated signaling by the "decoy" receptor CXCR7. *Proc. Natl. Acad. Sci. U.S.A.* **107**, 628–632 (2010).

43. L. A. Borthwick, J. Mcgaw, G. Conner, C. J. Taylor, V. Gerke, A. Mehta, L. Robson, R. Muimo, The formation of the cAMP/protein kinase A-dependent annexin 2–S100A10 complex with cystic fibrosis conductance regulator protein (CFTR) regulates CFTR channel function. *Mol. Biol. Cell* **18**, 3388–3397 (2007).

44. L. A. Borthwick, A. Neal, L. Hobson, V. Gerke, L. Robson, R. Muimo, The annexin 2-S100A10 complex and its association with TRPV6 is regulated by cAMP/PKA/CnA in airway and gut epithelia. *Cell Calcium* **44**, 147–157 (2008).

45. K. Okuse, M. Malik-Hall, M. D. Baker, W.-Y. L. Poon, H. Kong, M. V. Chao, J. N. Wood, Annexin II light chain regulates sensory neuron-specific sodium channel expression. *Nature* **417**, 653–656 (2002).

46. E. Donier, F. Rugiero, K. Okuse, J. N. Wood, Annexin II light chain p11 promotes functional expression of acid-sensing ion channel ASIC1a. *J. Biol. Chem.* **280**, 38666–38672 (2005).

47. T. Foulkes, M. A. Nassar, T. Lane, E. A. Matthews, M. D. Baker, V. Gerke, K. Okuse, A. H. Dickenson, J. N. Wood, Deletion of annexin 2 light chain p11 in nociceptors causes deficits in somatosensory coding and pain behavior. *J. Neurosci.* **26**, 10499–10507 (2006).

48. K. Doi, R. Kise, K. Shimizume, M. Yanagawa, A. Inoue, A luminescent calcium biosensor enabling endpoint measurement of GPCR-mediated calcium signaling. *Commun. Biol.* **9**, 695 (2026).

49. T. Lieu, E. Savage, P. Zhao, L. Edgington-Mitchell, N. Barlow, R. Bron, D. P. Poole, P. McLean, R. Lohman, D. P. Fairlie, N. W. Bunnett, Antagonism of the proinflammatory and pronociceptive actions of canonical and biased agonists of protease-activated receptor-2. *Br. J Pharmacol.* **173**, 2752–2765 (2016).

50. A. F. Paszcuk, N. L. M. Quintão, E. S. Fernandes, L. Juliano, K. Chapman, P. Andrade-Gordon, M. M. Campos, N. Vergnolle, J. B. Calixto, Mechanisms underlying the nociceptive and inflammatory responses induced by trypsin in the mouse paw. *Eur. J. Pharmacol.* **581**, 204–215 (2008).

51. N. Vergnolle, N. Cenac, C. Altier, L. Cellars, K. Chapman, G. Zamponi, S. Materazzi, R. Nassini, W. Liedtke, F. Cattaruzza, E. Grady, P. Geppetti, N. Bunnett, A role for transient receptor potential vanilloid 4 in tonicity-induced neurogenic inflammation. *Br. J Pharmacol.* **159**, 1161–1173 (2010).

52. S. Barbash, T. Persson, E. Lorenzen, M. A. Kazmi, T. Huber, T. P. Sakmar, Detection of concordance between transcriptional levels of GPCRs and receptor-activity-modifying proteins. *iScience* **11**, 366–374 (2019).
